# A Potential Role for the Amyloid Precursor Protein in the Regulation of Interferon Signaling, Cholesterol Homeostasis, and Tau Phosphorylation in Niemann–Pick Disease Type C

**DOI:** 10.3390/genes15081066

**Published:** 2024-08-13

**Authors:** Kayla L. Sanchez, Samuel D. Shin, Naren P. Rajagopal, Jacob B. White, Antonio Currais, David Soriano-Castell, Pamela Maher, Salvador Soriano

**Affiliations:** 1 Department of Pathology and Human Anatomy, School of Medicine, Loma Linda University, Loma Linda, CA 92354, USA; klsanchez@students.llu.edu (K.L.S.); shins2@ccf.org (S.D.S.); 1narenr@gmail.com (N.P.R.); jwhite01@students.llu.edu (J.B.W.); 2 The Salk Institute for Biological Studies, 10010 North Torrey Pines Road, La Jolla, CA 92037, USA; acurrais@salk.edu (A.C.); dsorianocastell@salk.edu (D.S.-C.)

**Keywords:** Niemann-Pick disease type C, Amyloid Precursor Protein, neurodegeneration

## Abstract

Niemann–Pick disease type C (NPC) is a rare and fatal neurological disorder caused by mutations in *Npc1* or *Npc2*, with *Npc1* accounting for 95% of cases. These mutations result in the functional loss of their respective proteins, causing cellular abnormalities characterized by disrupted lipid dysregulation, calcium dysfunction, elevated damage associated molecular patterns (DAMPs), and a pro-inflammatory environment. This cellular pathology ultimately triggers neurodegeneration, with the cerebellum being the earliest and most affected region. We have recently shown atypical activation of interferon signaling in the presymptomatic *Npc1*^−/−^ mouse cerebellum and, to a lesser extent, in the cerebral cortex. In addition, we reported that the Amyloid Precursor Protein (APP) is an NPC disease modifier. Loss of APP function leads to widespread neurodegeneration in the NPC brain, including exacerbated interferon signaling in the cerebellum. To better understand the role of APP as a disease modifier throughout the NPC brain, here we carried out a transcriptomic analysis of the cerebral cortex and cerebellum from 3-week-old *Npc1*^−/−^ mice as well as age-matched controls in the presence and absence of APP. We report differential effects of APP loss of function in the cerebral cortex and cerebellum, including cholesterol and tau dysregulation, in both brain regions. Our findings demonstrate a novel link between APP loss and early pathogenic mechanisms in NPC.

## 1. Introduction

Niemann–Pick disease type C (NPC) is a fatal disorder with a global incident rate of 1 in 120,000 individuals [1]. It is caused by mutations in the *Npc1* (Chr.18q11.2) or *Npc2* (Chr.14q24.3) genes that result in the functional loss of their corresponding proteins [2]. Although the exact functions of *Npc1* and *Npc2* proteins are not fully understood, cellular findings of NPC include lipid dysregulation [3,4,5], calcium dysfunction [3], elevated damage associated molecular patterns (DAMPs), mitochondrial dyshomeostasis, high levels of reactive oxygen species (ROS) and lipid peroxidation, and a pro-inflammatory environment [4,5], ultimately resulting in neuronal death.

NPC pathology is characterized by clear spatiotemporal patterns of neurodegeneration, and the cerebellum is the earliest and most severely affected region as compared with cerebral regions, with widespread loss of Purkinje neurons [6]. This pattern of pathology parallels the cerebellar symptoms observed in NPC patients [7,8]. The mechanisms leading to these differential patterns of neurodegeneration are not well understood. In that regard, we have recently shown in a mouse model of NPC that there is an atypical activation pattern of IFN-γ and IFN-α-signaling in the cerebellum at 3 weeks of age, before symptoms are apparent, and, to a lesser extent, in the cerebral cortex. Furthermore, we unveiled significant differences in the regulation of oxidative stress between the cerebellum and the cerebral cortex at this early, pre-symptomatic age, as well as in the cerebellum [4,7].

Here, we have evaluated the role of the Amyloid Precursor Protein (APP) in the NPC brain. Recently [5,9], we proposed that APP function is central to NPC pathogenesis, because of its role as the precursor of the amyloid peptide Aβ, which accumulates in the NPC brain. Aβ accumulation is also accompanied by the presence of tau neurofibrillary tangles (NFTs), and this is important because both hallmarks are also prominent in Alzheimer’s disease (AD) and, according to the amyloid cascade hypothesis of neurodegeneration, AD pathogenesis is driven by Aβ accumulation, which, in turn, leads to tau NFT formation and, ultimately, cell death [9,10]. In that context, in an attempt to dissect out the role of Aβ in NPC pathogenesis, we characterized the gross phenotypes of *Npc1* knockout (*Npc1*^−/−^/*App*^+/+^) and *Npc1*/*App* double knockout mice (*Npc1*^−/−^/*App*^−/−^) [9]; we reasoned that removing APP from the NPC brain could improve the disease phenotype because of the lack of Aβ and that this could unveil additional APP-specific mechanisms of neurodegeneration in NPC. Unexpectedly, we found that loss of APP in the NPC brain leads to a dramatic deterioration of the disease phenotype, including earlier disease onset and a shorter life-span, as well as exacerbation of cholesterol dysregulation, tau hyperphosphorylation, and inflammation [9]. In addition, APP loss exacerbates the pathogenic activation pattern of cerebellar IFN-γ and IFN-α-signaling [5].

In order to explore the mechanisms that lead to such a widespread deleterious impact of APP loss in the NPC brain, we carried out a wide-genome transcriptome analysis of cerebella and cerebral cortices from 3-week-old mice of the following *Npc1* and *App* genotypes: wildtype control (*Npc1*^+/+^/*App*^+/+^); *App* knockout (*Npc1*^+/+^/*App*^−/−^); *Npc1* knockout (*Npc1*^−/−^/*App*^+/+^); and *Npc1*/*App* double knockout (*Npc1*^−/−^/*App*^−/−^). We report that loss of APP function further increases the aberrant interferon patterns that occur early in the NPC brain and negatively impacts tau phosphorylation patterns and cholesterol homeostasis. APP loss impacts these pathways to different degrees in the cerebral cortex and cerebellum. We discuss the implications of our findings in the context of the spatio-temporal progression of pathology in NPC disease.

## 2. Materials and Methods

### 2.1. Animals

All experiments were approved by the Loma Linda University Institutional Animal Care and Use Committee (LLU#8170041 and LLU#8180006). Wildtype control (*Npc1*^+/+^/*App*^+/+^), *App* knockout (*Npc1*^+/+^/*App*^−/−^), *Npc1* knockout (*Npc1*^−/−^/*App*^+/+^), and *Npc1*/*App* double knockout (*Npc1*^−/−^/*App*^−/−^) mice were generated as previously described [5].

### 2.2. Transcriptome Analysis

Cerebral cortex and cerebellum samples from 3-week-old *Npc1*^+/+^/*App*^+/+^, *Npc1*^+/+^/*App*^−/−^, *Npc1*^−/−^/*App*^+/+^, and *Npc1*^−/−^/*App*^−/−^ *mice* were sent to GenUs (GenUs Biosystems, Northbrook, IL, USA) for analysis. RNA was extracted from the samples and purified using Ribopure (Ambion; Fisher Healthcare, Houston, TX, USA) RNA isolation. Total RNA samples were quantitated by UV spectrophotometry (OD260/280). The quality of the total RNA was then analyzed with an Agilent Bioanalyzer. After preparation of cDNA and cRNA strands, the cRNA was hybridized to Agilent Mouse v2 GE 4x44K arrays and then scanned on an Agilent G2565 Microarray Scanner (Aligent, Santa Clara, CA, USA). Data were analyzed with Agilent Feature Extraction and GeneSpring GX v7.3.1 software.

### 2.3. Data Preprocessing

Raw counts were normalized to the average expression of all genes. Microsoft Excel files provided by GenUs were converted to comma-separated value (CSV) files. The data were filtered to remove null values and duplicates. The data were then formatted in the form required for the differential expression tools.

### 2.4. Differential Gene Expression Analysis

Pre-processed data were analyzed for differential gene expression. To correct for multiple comparisons, the statistical significance of normalized expression levels between groups was determined by the Kruskal–Wallis one-way analysis of variance (ANOVA) and a protected Tukey post hoc test, with a *p*-value of less than 0.05 considered significant. Log-fold change was determined for each gene with the following comparisons: *Npc1*^−/−^ cerebral cortex vs. wildtype cortex, *Npc1*^−/−^/*App*^−/−^ cerebral cortex vs. wildtype cortex, *Npc1*^−/−^ cerebellum vs. wildtype cerebellum, and *Npc1*^−/−^/*App*^−/−^ cerebellum vs. wildtype cerebellum.

### 2.5. Ingenuity Pathway Analysis

Datasets with differentially expressed genes were imported into the Ingenuity Pathway Analysis (IPA, Qiagen, Redwood City, CA, USA) program and analyzed for a comprehensive look at differentially expressed pathways and ontologies. Genes with an expression fold change (FC) score between −1.5 and 1.5 were removed from the analysis. IPA scores of |Z| > 2 and a *p*-value <0.05 were considered significant.

### 2.6. Gene Set Enrichment Analysis

Gene set enrichment analysis was conducted using GSEA software V.4.3.2 (GSEA, www.broadinstitute.org/gsea, accessed on 7 February 2024) on significant DEGs. Gene set enrichment was run against REACTOME_TLR4 CASCADE, GOBP_RESPONSE TO ROS, REACTOME_DETOXIFIC-

ATION_OF_REACTIVE-OXYGEN_SPECIES, BIOCARTA_NFKB _PATHWAY, GOMF_

TAU_PROTEIN_KINASE_ACTIVITY, and GOBP_REGULATION_OF_TAU_PROTEIN_

KINASE_ACTIVITY. The parameters used in the GSEA were as follows: The permutation type for enrichment analysis was based on phenotype, and the number of permutations was 1000. The chip type was “Mouse_Ensembl_Gene_ID_MSigDB.v2023.1.Mm. chip” for the mapping of gene IDs. Gene sets with over 500 genes were excluded. The enrichment statistic was “weighted”, and the metric for ranking genes was “log2_Ratio_of_Classes.” The gene list sorting mode was “real”, and the gene list ordering was by “descending” order.

## 3. Results

### 3.1. Loss of APP Amplifies Interferon Signaling in the Npc1^−/−^ Cerebral Cortex and Cerebellum

**Figure 1:**
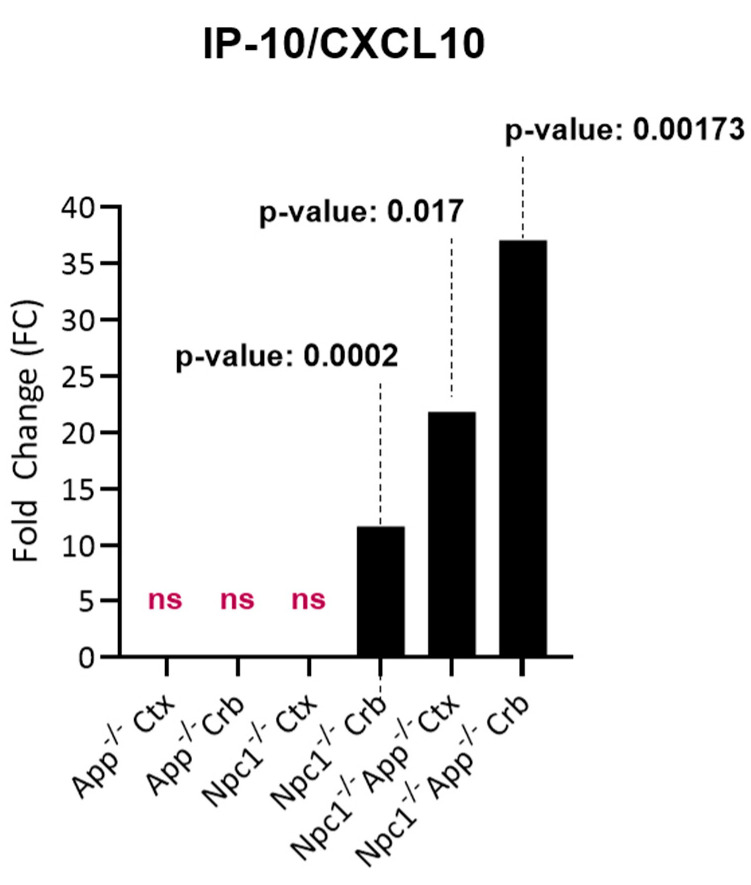
Fold change in IP-10/CXCL10 across genotypes. APP depletion increased expression of IP-10/CXCL10 in the NPC cerebral cortex and cerebellum. Significance is determined by an absolute fold change of aFC > 1.5 and a *p*-value < 0.05. ns: non-significant.

We have recently shown that IFN-γ and IFN-α signaling are the most prominent pathways upregulated in the pre-symptomatic *Npc1*^−/−^ cerebellum and, to a lesser extent, in the cerebral cortex [11]. Notably, the IFN-γ/IFN-α effector IP-10/CXCL10 was the only measurable cytokine at this stage in the cerebellum, suggesting an early-mediator role for this cytokine in NPC pathogenesis. A comparison of IP-10/CXCL10 gene expression levels in the cerebella and cerebral cortices of *Npc1*^−/−^, *App*^−/−^, and *Npc1*^−/−^/*App*^−/−^ genotypes (Figure 1) shows that IP-10/CXCL10 is not detectable in the cerebral cortex of *Npc1*^−/−^ and *App*^−/−^ mice, but there is a remarkable 11.72-fold increase in its expression in the *Npc1*^−/−^ cerebellum. Loss of APP in the NPC brain, both in the cerebral cortex and cerebellum, dramatically increases the levels of IP-10/CXCL10, with a much higher impact in the latter tissue. These findings are consistent with the notion that APP plays a protective role against early, IFN-driven inflammation in the NPC brain.

Next, we carried out a genome-wide transcriptome analysis of the cerebral cortex and cerebella from *Npc1*^−/−^, *App*^−/−^, and *Npc1*^−/−^/*App*^−/−^ genotypes. As shown in Table 1, *Npc1*^−/−^ cerebella displayed 387 significant (aFC > 1.5 and *p*-value < 0.05) differentially expressed genes (DEGs) versus the wildtype controls, of which 276 were upregulated and 211 were downregulated, whereas in the cerebral cortex, *Npc1*^−/−^ mice displayed 321 DEGs, of which 199 were upregulated and 122 were downregulated. Additional loss of APP in the cerebellum (*Npc1*^−/−^/*App*^−/−^) resulted in an increase in the number of significant DEGs to 1973, with 1265 upregulated and 708 downregulated, whereas in the cerebral cortex, there were 1884 genes that were differentially expressed, 1177 of them upregulated and 707 downregulated (Table 1). *App*^−/−^ mice also displayed an increase in DEGs, both in the cerebral cortex (843, of which 459 were upregulated and 384 were downregulated), and in the cerebellum (1065, of which 457 were upregulated and 608 were downregulated). 

**Figure 2:**
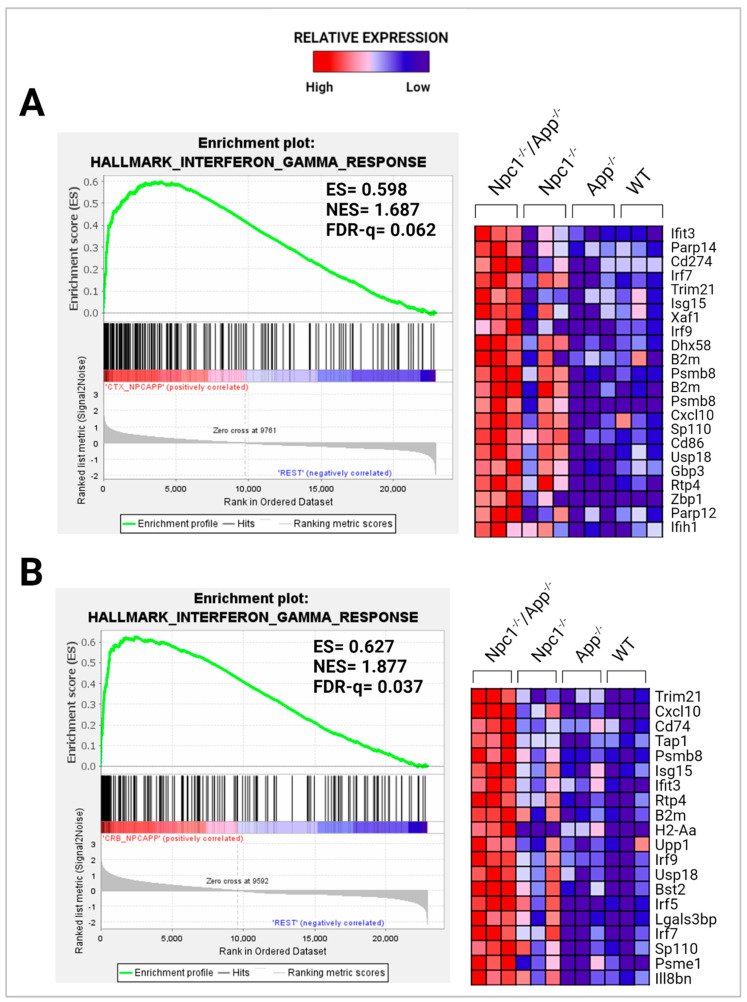
Activation of IFN-γ signaling in the *Npc1*^−/−^*App*^−/−^ cerebral cortex and cerebellum. Gene set enrichment analysis (GSEA) shows the activation of IFN-γ in the *Npc1*^−/−^*App*^−/−^ cerebral cortex (**A**) and cerebellum (**B**) when compared with its *Npc1*^−/−^/*App*^+/+^, *Npc1^+/+^/App*^−/−^, and *Npc1*^+/+^/*App*^+/+^ counterparts. ES = enrichment score; NES = normalized enrichment score; FDR-q = false discovery rate q-value. Significance is determined by an FDR-q < 0.25.

**Figure 3:**
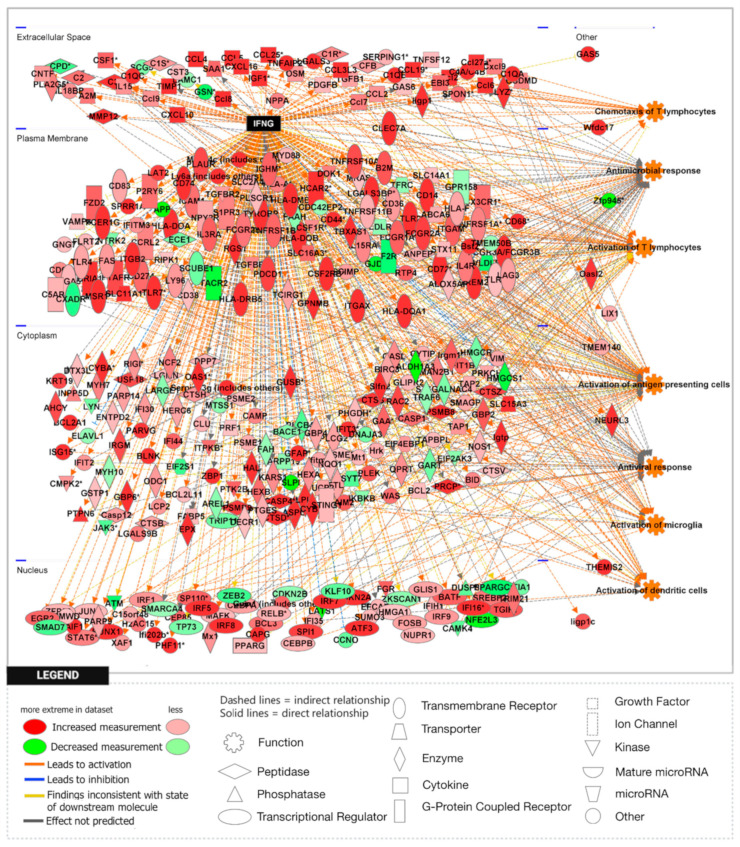
Activation of IFN-γ-responsive genes in the *Npc1*^−/−^*App*^−/−^ mouse cerebral cortex. Two hundred and eighty-six IFN-γ-responsive genes are differentially expressed in the *Npc1*^−/−^*App*^−/−^ cerebral cortex compared with age-matched wildtype littermates. Of those, two-hundred and twenty-nine DEGs are significantly upregulated and fifty-seven are significantly downregulated. All DEGs are displayed in their sub-cellular location. All DEGs meet the significance criteria for the absolute fold change (aFC > 1.5) and *p*-value (*p* < 0.05). (*) indicates a duplicate identifier that corresponds to a signle gene.

**Figure 4:**
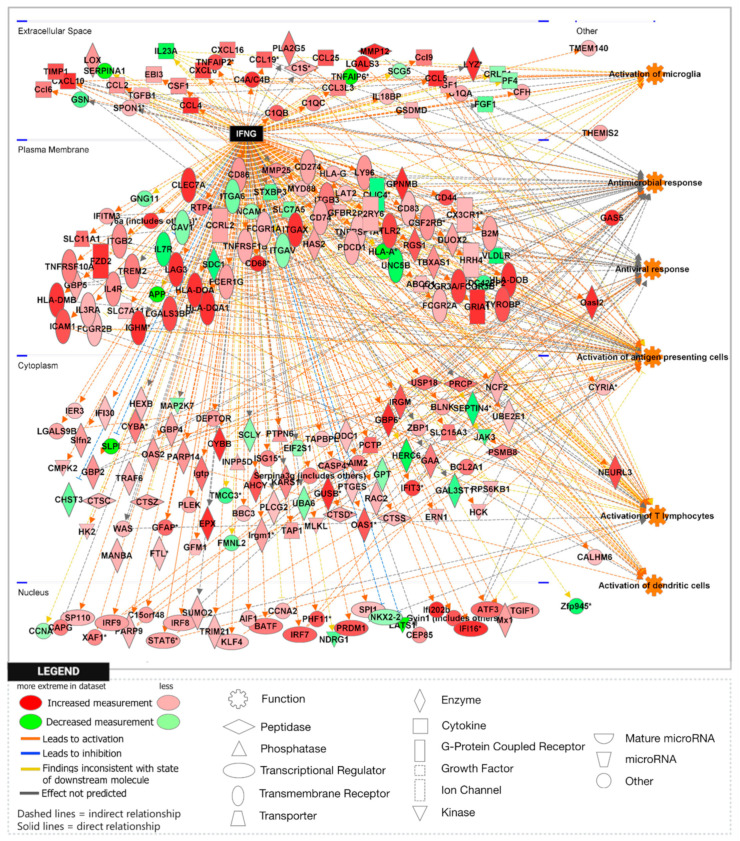
Activation of IFN-γ-responsive genes in the *Npc1*^−/−^*App*^−/−^ mouse cerebellum. Three hundred and fifty-eight IFN-γ-responsive genes are differentially expressed in the *Npc1*^−/−^*App*^−/−^ cerebellum compared with age-matched wildtype littermates. Of those, two-hundred and ninety-eight DEGs are significantly upregulated and sixty are significantly downregulated. All DEGs are displayed in their sub-cellular location. All DEGs meet the significance criteria for the absolute fold change (aFC > 1.5) and *p*-value (*p* < 0.05). (*) indicates a duplicate identifier that corresponds to a signle gene.

To further explore the significance of these findings, we performed gene set enrichment analysis (GSEA) using the H1 Hallmark database. GSEA hallmark gene sets are derived from the Molecular Signatures Database (MSigDB) and represent well-defined biological states or processes in which significance is noted by a false discovery rate (FDR)-q-value < 0.25. Notably, GSEA showed IFN-γ to be the most enriched gene set in the *Npc1*^−/−^/*App*^−/−^ cerebral cortex when compared with the remaining genotypes (Figure 2A,B), consistent with a protective role for APP against IFN-γ-driven inflammation. GSEA provides a broad overview by ranking genes within an expression dataset without contextualizing the molecular interactions amongst genes. To overcome this limitation, we employed Ingenuity Pathway Analysis (IPA) to examine how these genes interact within biological pathways. Consistent with our GSEA findings, IPA Upstream Analysis identified IFN-γ as one of the top transcriptional regulators in the *Npc1*^−/−^/*App*^−/−^ cerebral cortex versus wildtype controls, showing the activation of 201 related genes and a z-score of 8.494, higher than reported in the *Npc1*^−/−^ and *App*^−/−^ cerebral cortex (Table 2). This trend was also found in the *Npc1*^−/−^/*App*^−/−^ cerebella, which showed the activation of 262 IFN-γ related genes and a z-score of 9.324 versus wildtype controls (Table 3). Interestingly, no impact on IFN signaling was detected in *App*^−/−^ mice, despite the large number of DEGs (Table 1). Next, we used IPA Pathway Analysis to identify genes that are influenced by IFN-γ and IFN-α. As shown in Figure 3, the *Npc1*^−/−^/*App*^−/−^ cerebral cortex displayed 286 IFN-γ-responsive genes compared with the 84 DEGs we recently reported in the *Npc1*^−/−^ cerebral cortex [11] and 71 in the *App*^−/−^ cerebral cortex (Supplementary Figure S1). From the 286 *Npc1*^−/−^/*App*^−/−^ DEGs, IPA Disease and Function *Analysis* revealed the upregulation of the following processes (Table 4): microglial activation; antiviral response; antimicrobial response; T-lymphocyte activation; activation of antigen-presenting cells; and activation of dendritic cells. Depletion of the *App* gene also affected the *Npc1*^−/−^ cerebellum, with the identification of 358 IFN-γ-responsive genes (Figure 4) compared with the 60 DEGs we recently reported in the *Npc1*^−/−^ cerebellum. From the 358 *Npc1*^−/−^/*App*^−/−^ DEGs, IPA Disease and Function Analysis revealed the upregulation of the following processes (Table 4): microglial activation; antiviral response; antimicrobial response; T-lymphocyte activation; T-lymphocyte chemotaxis; activation of antigen-presenting cells, and activation of dendritic cells (Figure 4, Table 4). Thus, loss of APP in both *Npc1*^−/−^ tissues led to an exacerbation of IFN-γ-responsive genes and the activation of additional pathogenic pathways.

**Figure 5:**
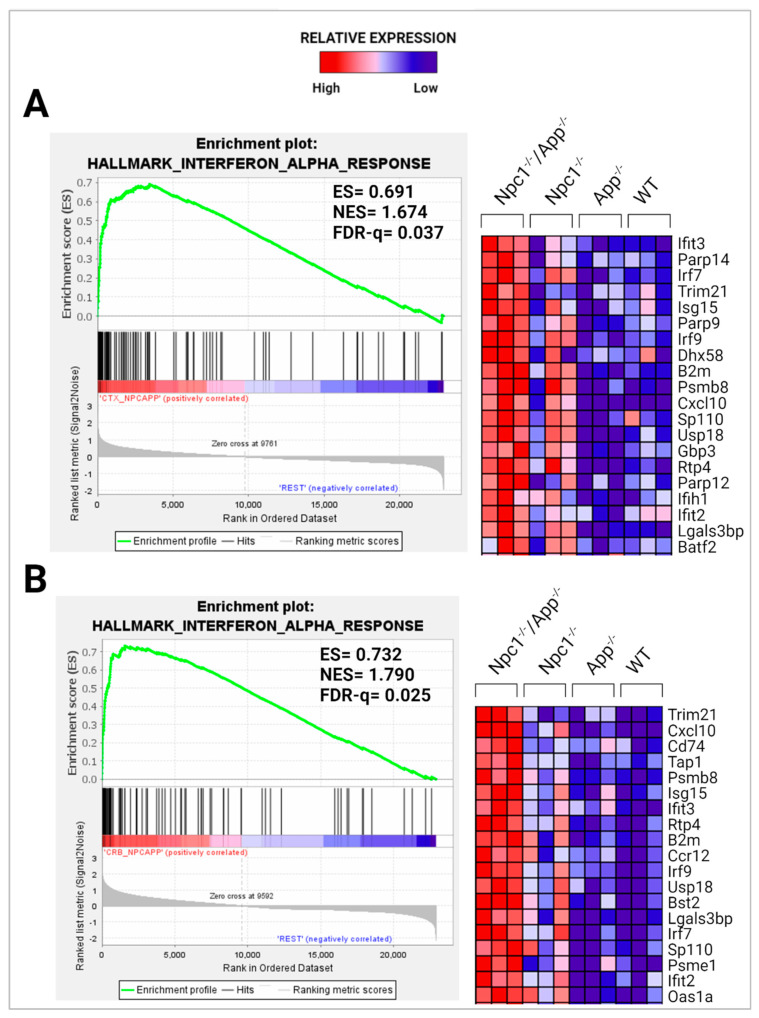
Activation of IFN-α signaling in the *Npc1*^−/−^*App*^−/−^ cerebral cortex and cerebellum. Gene set enrichment analysis (GSEA) shows activation of IFN-α in the *Npc1*^−/−^*App*^−/−^ cerebral cortex (**A**) and cerebellum (**B**) when compared with its *Npc1*^−/−^/*App*^+/+^, *Npc1^+/+^/App*^−/−^, and *Npc1*^+/+^/*App*^+/+^ counterparts. ES = enrichment score; NES = normalized enrichment score; FDR-q = false discovery rate q-value. Significance is determined by an FDR-q < 0.25.

**Figure 6:**
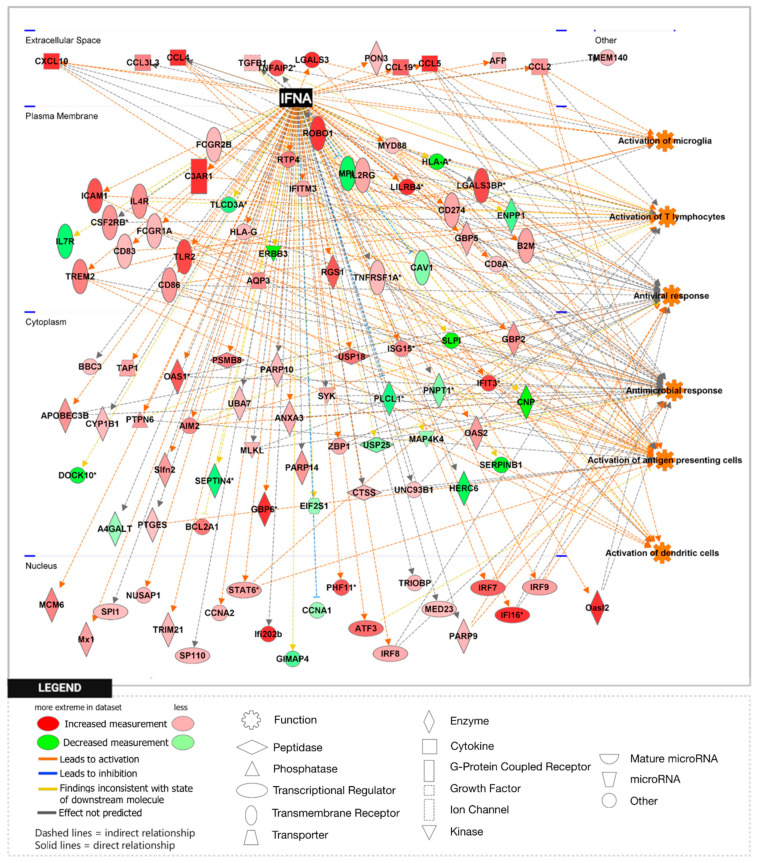
Activation of IFN-α-responsive genes in the *Npc1*^−/−^*App*^−/−^ mouse cerebral cortex. One hundred and twenty IFN-α-responsive genes are differentially expressed in the *Npc1*^−/−^*App*^−/−^ cerebral cortex compared with age-matched wildtype littermates. Of those, ninety-nine DEGs are significantly upregulated and twenty-one are significantly downregulated. All DEGs are displayed in their sub-cellular location. All DEGs meet the significance criteria for absolute fold change (aFC > 1.5) and *p*-value (*p* < 0.05). (*) indicates a duplicate identifier that corresponds to a signle gene.

**Figure 7:**
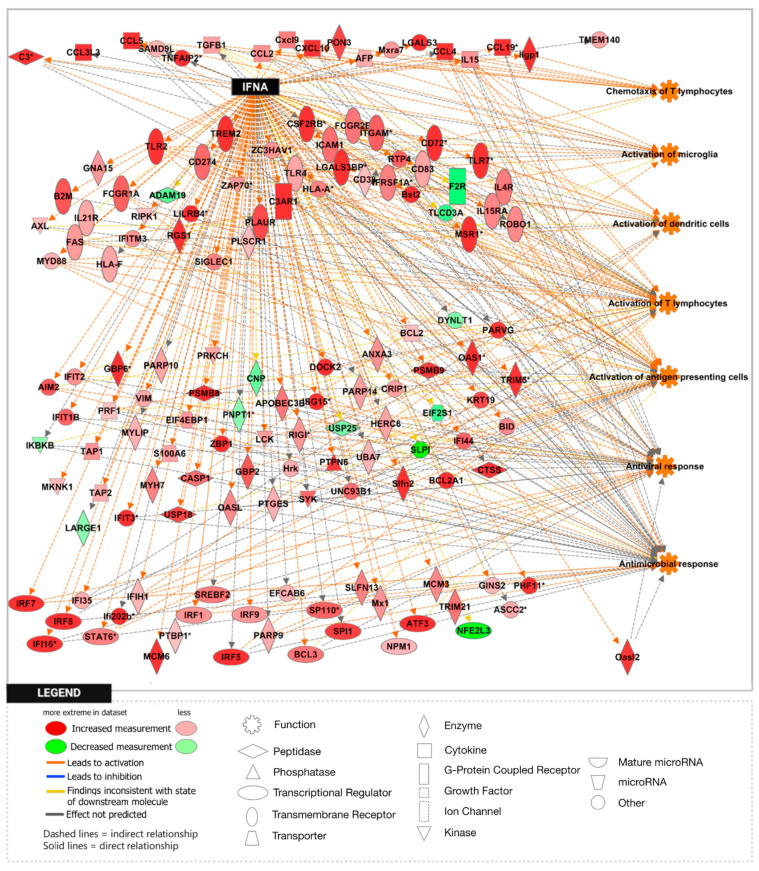
Activation of IFN-α-responsive genes in the *Npc1*^−/−^*App*^−/−^ mouse cerebellum. One hundred and forty-five IFN-α-responsive genes are differentially expressed in the *Npc1*^−/−^*App*^−/−^ cerebellum compared with age-matched wildtype littermates. Of those, one hundred and thirty-three DEGs are significantly upregulated and twelve are significantly downregulated. All DEGs are displayed in their sub-cellular location. All DEGs meet the significance criteria for absolute fold change (aFC > 1.5) and *p*-value (*p* < 0.05). (*) indicates a duplicate identifier that corresponds to a signle gene.

Interferon-α emerged as another highly enriched gene set in the *Npc1*^−/−^/*App*^−/−^ cerebral cortex in the H1 hallmark database (Supplementary Table S1). Figure 5 further shows that both brain regions from *Npc1*^−/−^/*App*^−/−^ mice displayed enrichment when compared with all other genotypes (Figure 5). These IFN-α-responsive genes also influence the expression of the downstream functions reported in Table 2 and Table 3 (Figure 6 and Figure 7). Distinct differences were also evident in the expression of several pro-inflammatory cytokines. In the *Npc1*^−/−^/*App*^−/−^ cerebral cortex, *Ccl2*, *Ccl4*, *Ccl5*, *Ccl6*, *Ccl9*, *Ccl21*, *Csf1*, *Cxcl6*, *Cxcl10*, and *Ebi3* were further upregulated, while *Cklf*, *Cxcl3*, *Cxcl12*, *Ccl28*, *Il9*, and *Spred2* were present only in the *Npc1*^−/−^ cerebral cortex (Supplementary Table S3). Overall, these data suggest that not only do levels of interferon-mediated inflammatory responses vary between brain regions, but they also differ in their reaction to APP loss.

### 3.2. APP Depletion Exacerbates Inflammatory Pathways Linked To DAMP Generation, ROS, and Lipid Peroxidation in the Npc1^−/−^ Cerebral Cortex

Our next aim was to investigate early pathogenic alterations that might underlie the observed increase in interferon-mediated inflammation in both the cerebral cortex and cerebellum in *Npc1*^−/−^*App*^−/−^ mice. In that regard, damage-associated molecular patterns (DAMPs), which are prevalent in NPC, as well as reactive oxygen species (ROS) production and lipid peroxidation, are likely candidates as initiators of IFN-mediated inflammation [12,13,14,15]. DAMPs exacerbate inflammation through binding to Toll-like receptors (TLRs), and TLR4 is of particular relevance here, because it accumulates in NPC endolysosomal compartments and drives NF-κB expression [16,17]. Therefore, we measured the impact of APP loss on TLR4 and NF-κB in the cerebral cortex and cerebellum of *Npc1*^−/−^ mice.

**Figure 8:**
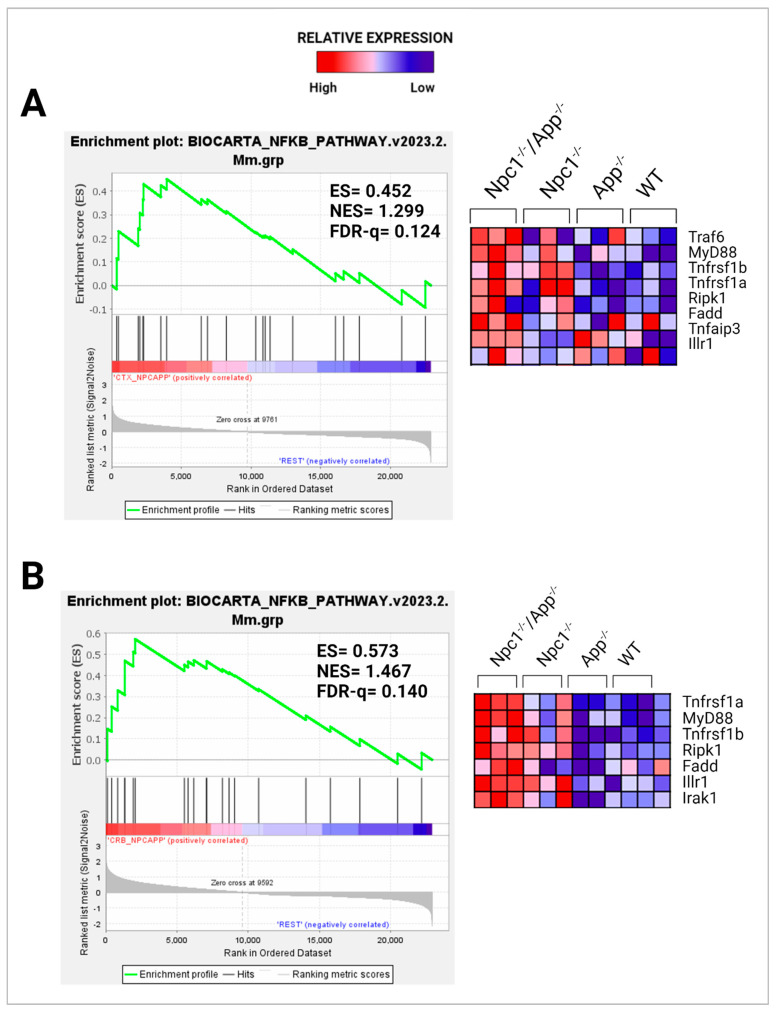
GSEA analysis of the NF-κB signaling pathway. Enrichment plot and heatmap showing significant enrichment in the (**A**) *Npc1*^−/−^*App*^−/−^ cerebral cortex and (**B**) cerebellum compared with its *Npc1*^−/−^/*App*^+/+^, *Npc1^+/+^/App*^−/−^, and *Npc1*^+/+^/*App*^+/+^ counterparts. ES = enrichment score; NES = normalized enrichment score; FDR-q = false discovery rate q-value. Significance is determined by an FDR-q < 0.25.

**Figure 9:**
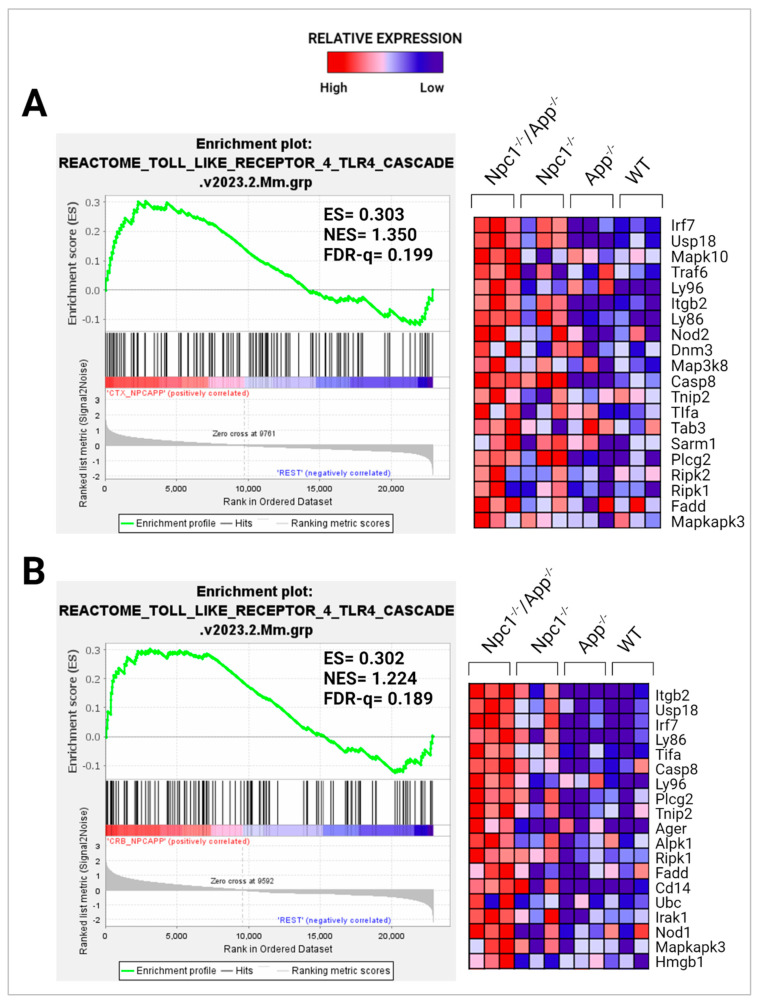
Activation of the Toll-like Receptor 4 (TLR4) cascade. GSEA TLR4 contains genes associated with the binding, trafficking, and processing of TLR4. Enrichment plot and heatmap showing significant enrichment in the (**A**) *Npc1*^−/−^/*App*^−/−^ cerebral cortex and (**B**) cerebellum when compared with its *Npc1*^−/−^/*App*^+/+^, *Npc1*^+/+^/*App*^−/−^, and *Npc1*^+/+^/*App*^+/+^ counterparts. ES = enrichment score; NES = normalized enrichment score; FDR-q = false discovery rate q-value. Significance is determined by an FDR-q < 0.25.

We have recently reported gene set enrichment of NF-κB and TLR4 pathways in the *Npc1*^−/−^ cerebella but not in the *Npc1*^−/−^ cerebral cortex when compared with wildtype controls [11]. As shown in Figure 8 and Figure 9, loss of APP in the *Npc1*^−/−^ brain led to increased activation of both pathways in both regions, again, consistent with a cytoprotective role of APP against these pathways in the brain.

Next, because cGAS-STING signaling is a driver of type I interferon response in the cerebellum of late-stage *Npc1*^−/−^ mice [18], we evaluated the c-GAS-STING pathway using IPA analysis in our pre-symptomatic mice. As shown in Supplementary Figure S9, there were no differences in the activation of this pathway across genotypes and tissues in our mice.

**Figure 10:**
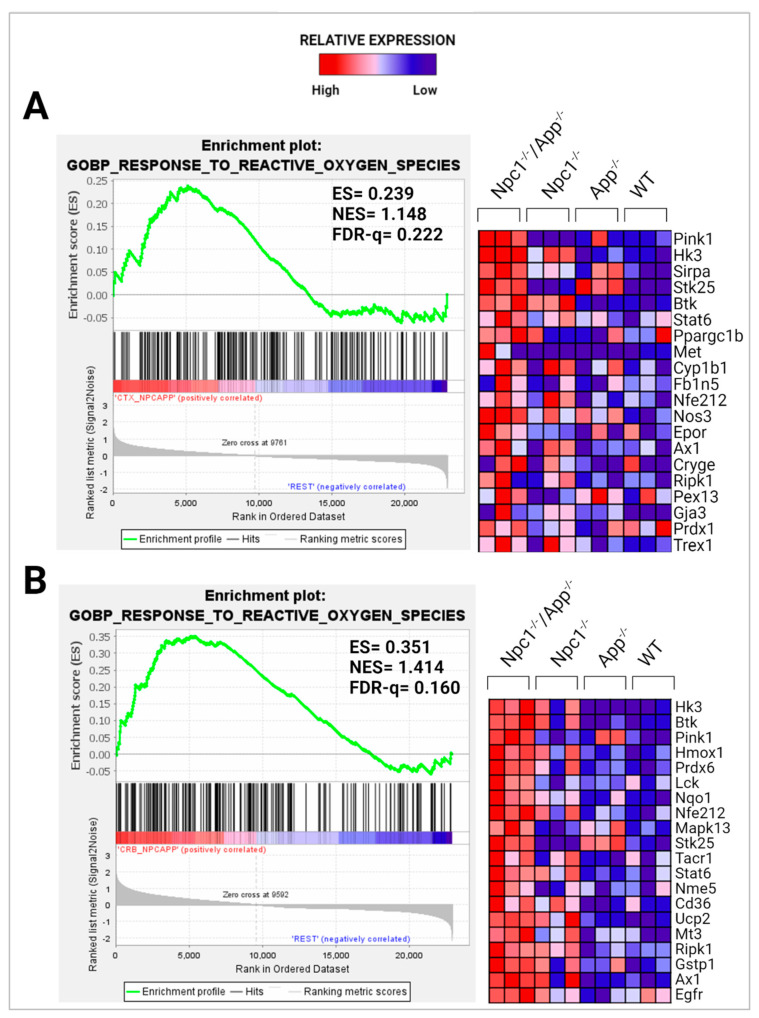
GSEA of the GOBP Response to ROS pathway. The Response to ROS pathway is enriched in the (**A**) *Npc1*^−/−^/*App*^−/−^ cerebral cortex and (**B**) cerebellum when compared with its *Npc1*^−/−^/*App*^+/+^, *Npc1*^+/+^/*App*^−/−^*,* and *Npc1*^+/+^/*App*^+/+^ counterparts. ES = enrichment score; NES = normalized enrichment score; FDR-q = false discovery rate q-value. Significance is determined by an FDR-q < 0.25.

In addition, we have shown, using GSEA analysis, that the GOBP Response to Reactive Oxygen Species pathway is significantly enriched in the *Npc1*^−/−^ cerebellum, compared with wildtype controls (NES = 1.245, FDR-q = 0.184) but not in the *Npc1*^−/−^ cerebral cortex (NES = 1.121, FDR-q = 0.276) [11]. To measure the impact of APP loss on this pathway, we carried out the same analysis in the *Npc1*^−/−^/*App*^−/−^ brain. As shown in Figure 10, loss of APP led to enrichment of this pathway in both brain regions. 

**Figure 11:**
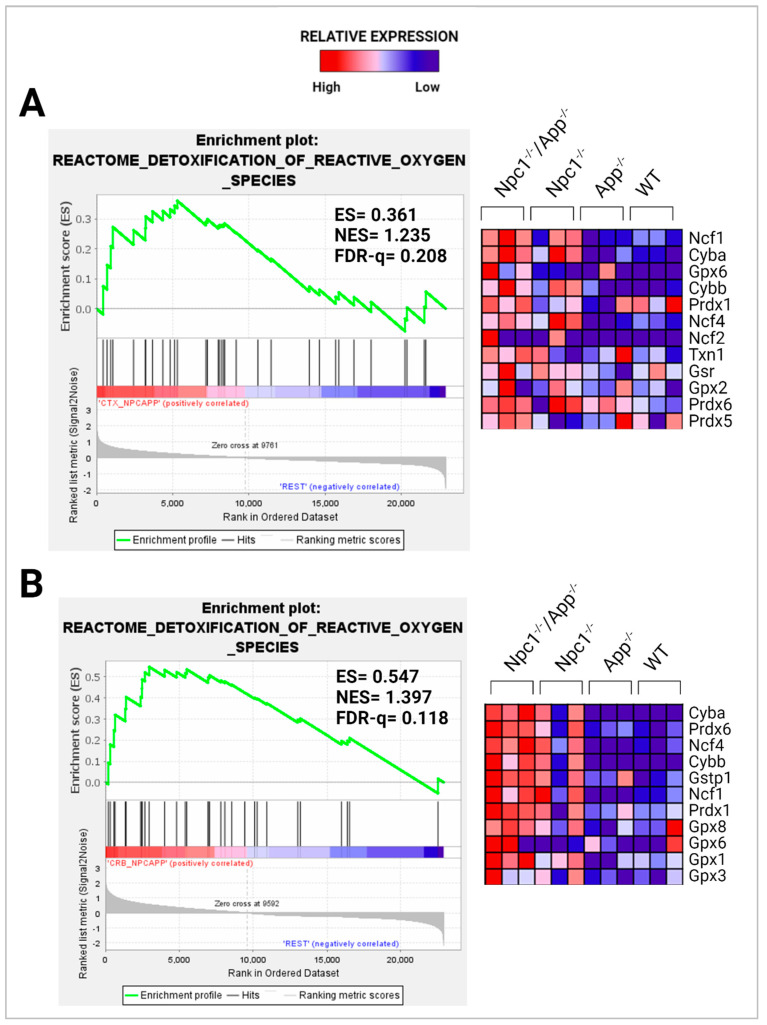
Detoxification of ROS. Gene set enrichment analysis of the Reactome Detoxification of Reactive Oxygen Species pathway. The Detoxification of Reactive Oxygen Species gene set is enriched in the (**A**) *Npc1*^−/−^/*App*^−/−^ cerebral cortex and (**B**) cerebellum when compared to its *Npc1*^−/−^/*App*^+/+^, *Npc1*^+/+^/*App*^−/−^, and *Npc1*^+/+^/*App*^+/+^, counterparts. ES = enrichment score; NES = normalized enrichment score; FDR-q = false discovery rate q-value. Significance is determined by an FDR-q < 0.25.

Our previous study revealed that the Detoxification of Reactive Oxygen Species gene set was significantly enriched in the *Npc1*^−/−^ cortex when compared to wildtype controls (FDR-q = 0.149) but not in the *Npc1*^−/−^ cerebellum [11]. By contrast, in the present analysis of the *Npc1*^−/−^/*App*^−/−^ brain, we observed enrichment of the Detoxification of ROS gene set in both the cerebral cortex and cerebellum when compared with the remaining genotypes (Figure 11A,B).

### 3.3. The Cellular Hormetic Response to 27-Hydroxycholesterol Is Impaired Following APP Depletion in the NPC Brain

**Figure 12:**
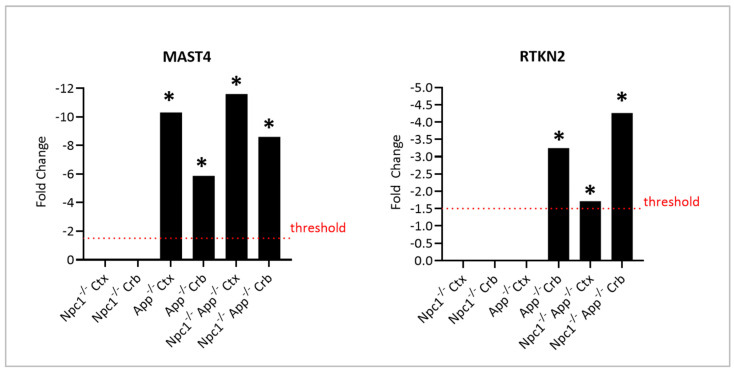
MAST4 and RTKN2 expression comparison in different genotypes and tissues. The bar graph displays the mRNA expression of MAST4 and RTKN2 against age-matched wildtype littermates. The fold-change threshold is characterized by the dotted line at |FC| > 1.5. The asterisk (*) denotes a *p*-value < 0.05.

We have previously reported severe dysregulation of cholesterol in the *Npc1*^−/−^/*App*^−/−^ mouse brain [9]. Subsequently, we showed that APP provides hormetic cytoprotection in the brain against 27-hydroxycholesterol (27OHC), a cholesterol derivative that binds APP and that is associated with neurodegeneration both in NPC and in AD [19], with a mechanism involving the modulation by APP of Rhotekin 2 (RTKN2), an NF-κB-dependent apoptotic regulator, and MAST4, a microtubule-associated kinase. Because the full functions of RTKN2 and MAST4 are not well understood, there are no suitably curated pathways for in silico analysis in the context of our current work. Interestingly, however, we found that while the expression of neither gene was significantly altered in the NPC brain (Figure 12), additional loss of APP led to dramatic changes in the expression of both genes in both the cerebellum and the cerebral cortex.

### 3.4. Npc1^−/−^ Tau Hyperphosphorylation Is Exacerbated by APP Depletion

**Figure 13:**
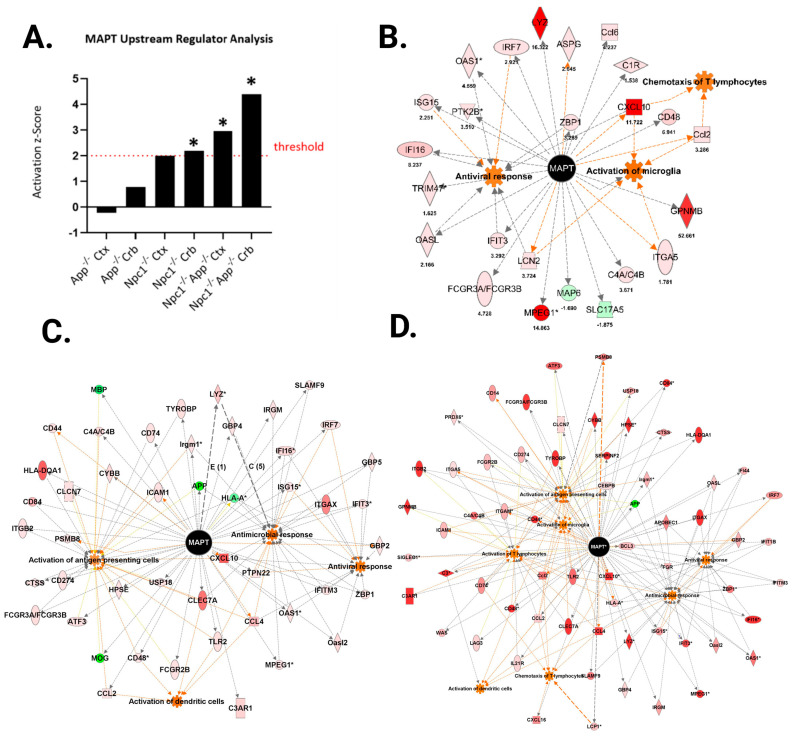
IPA Upstream Regulator Analysis (URA). (**A**) The URA identified Microtubule Associated Protein Tau (MAPT) as a significant upstream regulator in the *Npc1*^−/−^ cerebellum and both *Npc1*^−/−^*App*^−/−^ brain regions. Functional overlap maps were created for the three MAPT significant regions (**B**–**D**). (**B**). Tau is predicted to be an upstream regulator of genes contributing to the antiviral response, chemotaxis of T-lymphocytes, and microglial activation in the *Npc1*^−/−^ cerebellum. (**C**). MAPT in the *Npc1*^−/−^/*App*^−/−^ cerebral cortex contributes to the antiviral response, antimicrobial response, activation of dendritic cells, and activation of antigen-presenting cells. (**D**). The *Npc1*^−/−^/*App*^−/−^ cerebellum expressed more tau-regulated gene sets, activating the antiviral response, antimicrobial response, microglia, dendritic cells, antigen-presenting cells, and chemotaxis of T-lymphocytes. Upstream regulators (*) were determined through the overlap of dataset genes and known targets (overlap *p*-value < 0.05) and an absolute z-score ≥ 2.

Tau hyperphosphorylation is a pivotal event in the progression of many neurodegenerative disorders, including NPC [9,19]. Under physiological conditions, tau serves as a stabilizing factor for neuronal microtubules. However, long-term dysregulation of its phosphorylation patterns ultimately leads to its aggregation into neurofibrillary tangles [20]. We have previously shown aberrant tau phosphorylation patterns in the *Npc1*^−/−^/*App*^−/−^ brain, marked by an increase in pSer262/-356 (12E8), pSer202/T205 (AT8), and pSer396/404 (PHF-1) tau epitopes [9]. Those findings suggest that APP may function as a tau modulator independently of its role as the precursor of Aβ. Here, to explore the mechanisms involved, we carried out an IPA Upstream Regulator Analysis, which identified the pathway “Microtubule-Associated Protein Tau” as a significant upstream regulator in the *Npc1*^−/−^ cerebellum (Figure 13A), and the pathway was further activated in both brain regions in the *Npc1*^−/−^/*App*^−/−^ mice, suggesting that early-stage tau dysregulation may contribute to NPC pathogenesis. Interestingly, we found that MAPT-regulated genes also contributed to the activation of inflammatory and immune functions, as seen in Figure 13B–D. The *Npc1*^−/−^/*App*^−/−^ cerebellum showed the most MAPT-dependent alterations, with the microglia, antiviral response, antimicrobial response, antigen-presenting cells, dendritic cells, T-lymphocytes, and chemotaxis of T-lymphocytes all being activated (Figure 13D).

**Figure 14:**
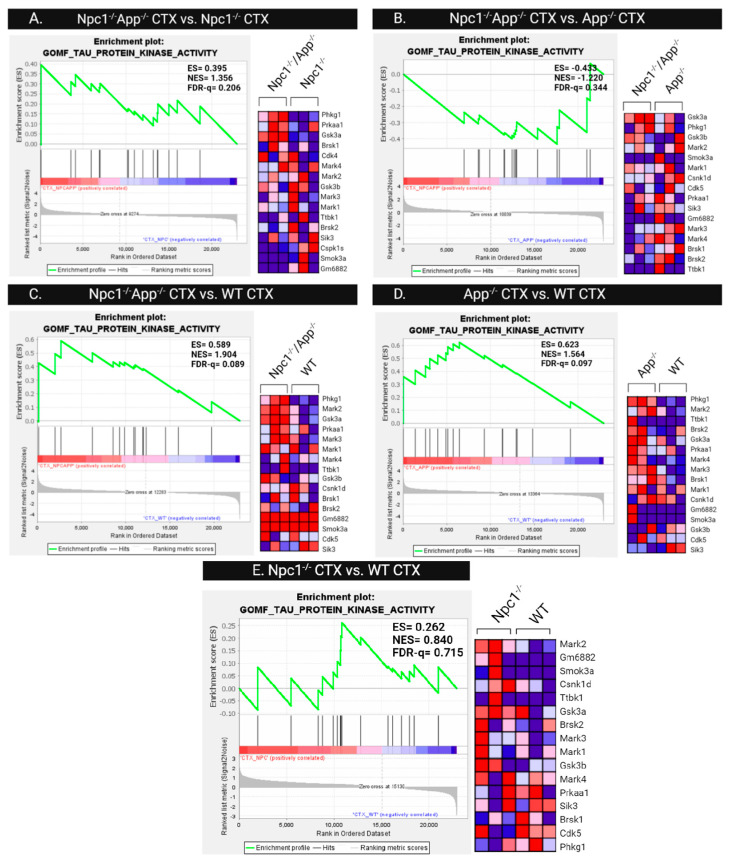
Tau protein kinase activity in the cerebral cortex. Gene set enrichment analysis of tau protein kinase activity is enriched in *Npc1*^−/−^/*App*^−/−^ when compared with *Npc1*^−/−^/*App*^+/+^ and *Npc1*^+/+^/*App*^+/+^ cerebral cortices (**A**,**C**). There were no differences between the *Npc1*^−/−^/*App*^−/−^ and *App*^−/−^ cerebral cortices (**B**) or *Npc1*^−/−^/*App*^+/+^ and *Npc1*^+/+^/*App*^+/+^ cerebral cortices (**E**), suggesting that APP loss alone influences tau kinase activity (**B**,**D**). ES = enrichment score; NES = normalized enrichment score; FDR-q = false discovery rate q-value. Significance is determined by an FDR-q < 0.25.

**Figure 15:**
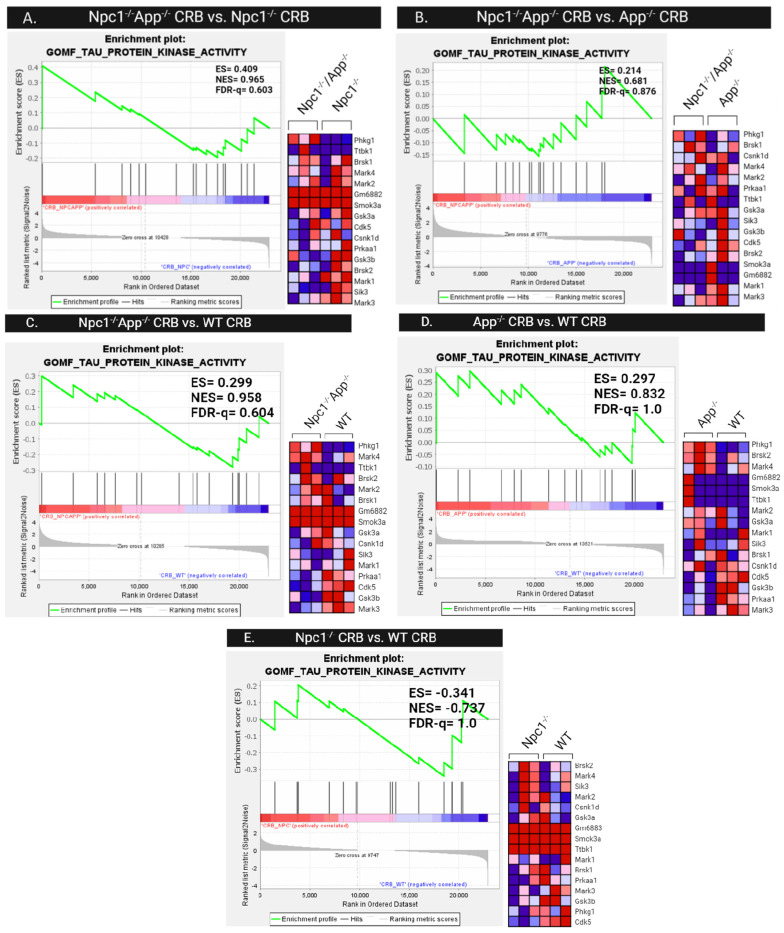
Tau Protein Kinase Activity in the Cerebellum. Gene set enrichment analysis of Tau Protein Kinase Activity is not present in any of the cerebellar genotypes as shown in figures (**A**–**E**). ES = enrichment score, NES = normalized enrichment score, FDR-q = false discovery rate q-value. Significance is determined by an FDR-q < 0.25.

Since post-translational modifications are the primary contributor to tau-related pathology, we next investigated differences in tau protein kinase activity patterns. Gene set enrichment analysis of the GOMF Tau Protein Kinase Activity gene set was performed across all cerebral cortical (Figure 14) and cerebellar (Figure 15) genotypes. Comparison of cerebral cortex samples of *Npc1*^−/−^/*App*^−/−^ and *Npc1*^−/−^ genotypes shows enrichment of tau protein kinase activity in *Npc1*^−/−^/*App*^−/−^ mice, with an NES = 1.356 and FDR-q = 0.206 (Figure 14). The cerebral cortex from both *Npc1*^−/−^/*App*^−/−^ and *App*^−/−^ mice also showed significant enrichment when compared with wildtype controls (Figure 14C,D). However, direct comparison between *Npc1*^−/−^/*App*^−/−^ and *App*^−/−^ cerebral cortices showed no difference in their enrichment of tau kinase activity (NES = −1.220, FDR-q = 0.344; Figure 14B). Enrichment of tau kinase activity was not present in any of the cerebellar genotypes (Figure 15).

**Figure 16:**
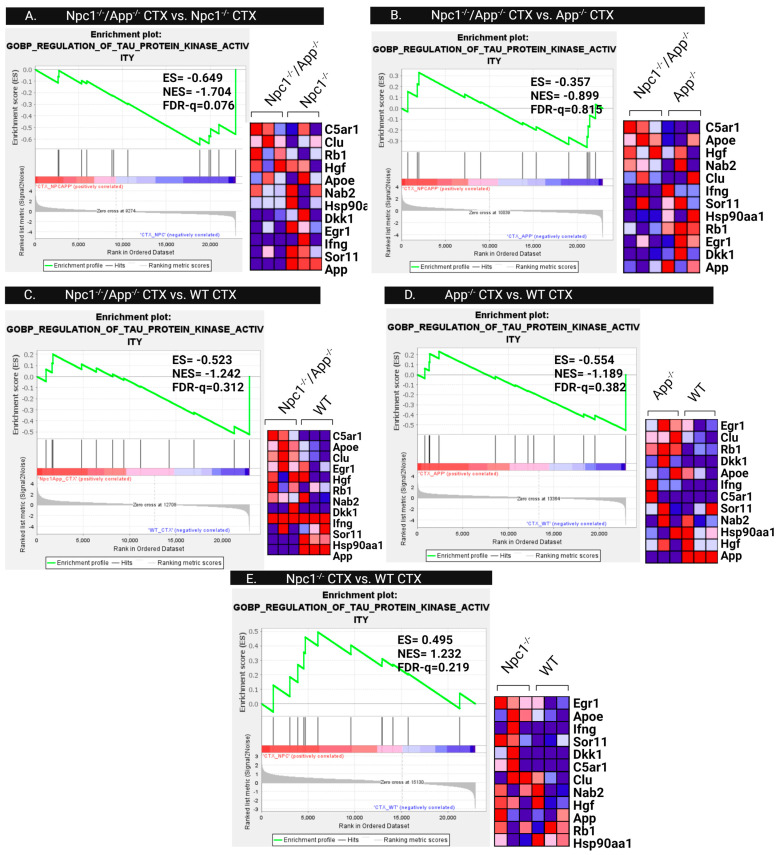
Regulation of Tau Protein Kinase Activity in the Cerebral Cortex. GSEA Regulation of Tau Protein Kinase Activity consistently showed enrichment in only the *Npc1*^−/−^ cerebral cortex (**A**,**E**). Suggesting that regulatory mechanisms are not disrupted in this tissue. (**B**–**D**) show no significant enrichment. ES = enrichment score, NES = normalized enrichment score, FDR-q = false discovery rate q-value. Significance is determined by an FDR-q < 0.25.

**Figure 17:**
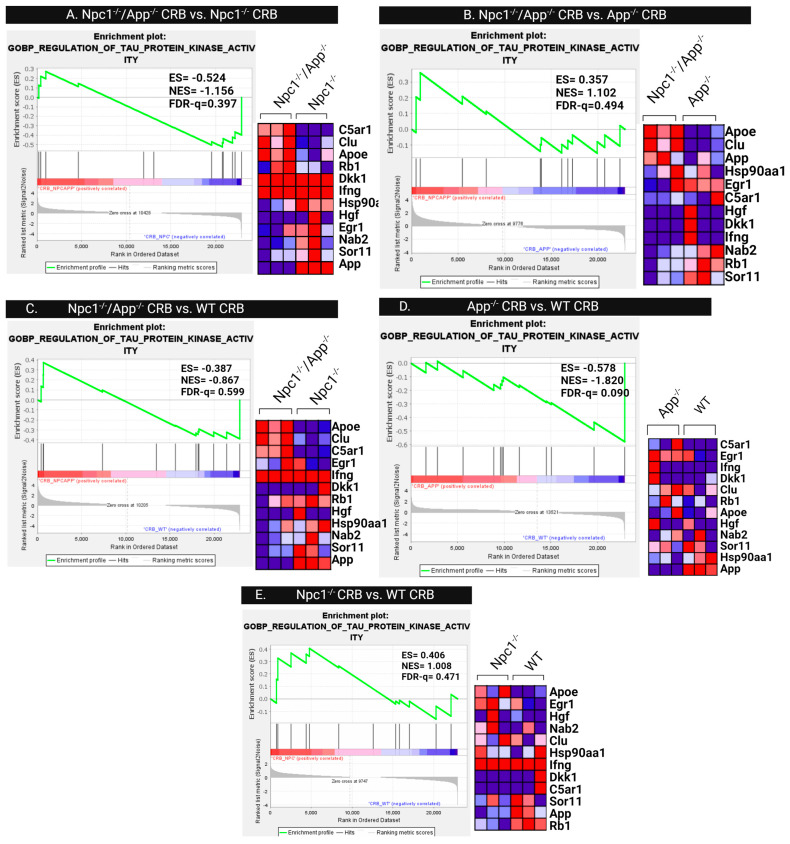
Regulation of tau protein kinase activity in the cerebellum. GSEA of the Regulation of Tau Protein Kinase Activity pathway showed no enrichment in any of the cerebella as reported in figures (**A**–**E**). ES = enrichment score; NES = normalized enrichment score; FDR-q = false discovery rate q-value. Significance is determined by an FDR-q < 0.25.

To further investigate the mechanisms involved in the observed tau kinase activity patterns of *Npc1*^−/−^/*App*^−/−^ mice, we next measured differences in the gene sets involved in tau kinase regulatory mechanisms, specifically analyzing the GOBP Regulation of Tau Protein Kinase Activity gene set. As seen in Figure 16, this gene set is enriched in the *Npc1*^−/−^ cerebral cortex (Figure 16E), and the enrichment is further increased in *Npc1*^−/−^/*App*^−/−^ mice when compared with *Npc1*^−/−^ mice (Figure 16A). Notably, analysis of cerebellar samples identified enrichment of the GOBP Regulation of Tau Protein Kinase Activity gene set, but we only found enrichment of this gene set in *App*^−/−^ mice (Figure 17D).

## 4. Discussion

Our genome-wide comparative analyses of transcriptomes of wildtype, *Npc1*^−/−^ and *Npc1*^−/−^/*App*^−/−^ genotypes from the cerebella and cerebral cortices of 3-week-old mice showed that loss of APP in the NPC brain leads to the exacerbation of multiple pathogenic pathways in both brain regions. It is important to note that APP in a wildtype genotype background appears to be dispensable, as evaluated by the absence of any over-phenotype in *App*^−/−^ mice, which have normal appearance, are fertile, and have a life-span identical to that of control mice [9]. In addition, as shown in Figure 1, Table 2 and Table 3, and Supplementary Figures S7 and S8, there are also no changes in IFN-driven pathways in the *App*^−/−^ mice. Consequently, we conclude that the transcriptomic changes seen in response to the loss of APP in the healthy wildtype brain do not have overt functional consequences. This conclusion is consistent with our view, detailed in references [10,18], of APP as a hormetic molecule against the dysregulation of cholesterol, with likely downstream effects on tau homeostasis and inflammation; since *App*^−/−^ mice are not exposed to additional stress stimuli, any functional consequences of APP loss (i.e., as seen in Supplementary Tables S2 and S3, Supplementary Figures S1–S4, S7 and S8) must be modest, as they do not translate into measurable phenotypic changes, at least within the age window of our experiments.

A striking finding in our analysis was the ~37-fold upregulation of the IFN-driven IP-10/CXCL10 cytokine in *Npc1*^−/−^/*App*^−/−^ cerebella (Figure 1), as well as the upregulation of IFN-γ and IFN-α signaling, when compared to *Npc1*^−/−^ brains [4,5] (Figure 2, Figure 3, Figure 4, Figure 5, Figure 6 and Figure 7). It is noteworthy that IP-10/CXCL10 was not detected in the *Npc1*^−/−^ cerebral cortex, whereas the additional loss of APP resulted in a remarkable ~21-fold increase (Figure 1; Table 4). It is conceivable that the increase in IP-10/CXCL10 and related immune responses in the *Npc1*^−/−^/*App*^−/−^ mice may be causative factors of the exacerbated phenotype of these mice [11].

In considering a possible origin for the IFN and IP-10/CXCL10 abnormalities in the NPC brain, we have suggested that the widespread presence of DAMPs, excessive generation of ROS, and lipid peroxidation, which are prominent in NPC, could be triggering sterile inflammatory patterns through NF-κB and Toll-like-receptor signaling pathways [11]. Our results here support the argument that loss of APP function in the NPC brain leads to further dysregulation of both pathways, both in the cerebral cortex and the cerebellum (Figure 8 and Figure 9) [9]. In addition, our analyses of the GOBP Response to Reactive Oxygen Species and the Detoxification of Reactive Oxygen Species gene sets sheds additional light on the role of APP on the modulation of oxidative stress in the NPC brain. While the GOBP Response to Reactive Oxygen Species gene set is significantly enriched in the *Npc1*^−/−^ cerebellum (NES = 1.245, FDR-q = 0.184) but not in the *Npc1*^−/−^ cerebral cortex (NES = 1.121, FDR-q = 0.276) [11], this gene set is enriched in both tissues in the *Npc1*^−/−^/*App*^−/−^ brain (Figure 10). Similarly, the Detoxification of Reactive Oxygen Species gene set is significantly enriched in the *Npc1*^−/−^ cortex (FDR-q = 0.149) but not in the *Npc1*^−/−^ cerebellum [11], whereas we observed enrichment of this gene set in both the cerebral cortex and cerebellum in the *Npc1*^−/−^/*App*^−/−^ brain (Figure 11A,B). Our interpretation of these findings is that the widespread increase in inflammatory-driven pathways in the *Npc1*^−/−^/*App*^−/−^ brain (Table 2, Table 3 and Table 4, Figure 2, Figure 3, Figure 4, Figure 5, Figure 6, Figure 7, Figure 8 and Figure 9) elicits a significant, but ultimately unsuccessful, activation of pathways in response to the increased oxidative stress in these mice, at least as evaluated by the enrichment scores of these gene sets.

We also observed upregulation of MAST4 and RTKN2 in *Npc1*^−/−^/*App*^−/−^ mice (Figure 12). These findings are significant because APP regulates cholesterol homeostasis through a pathway involving both MAST4 and RTKN2 [19], and gross phenotype characterization of the *Npc1*^−/−^/*App*^−/−^ mice shows an exacerbation of the cholesterol dysregulation seen in the *Npc1* brain [9]. Thus, it is plausible that loss of APP/MAST4/RTKN2-driven modulation of cholesterol in the *Npc1*^−/−^/*App*^−/−^ brain contributes to the dramatic exacerbation of cholesterol abnormalities seen in these mice [9,18]. In addition, because RTKN2 is NF-κB-dependent, and NF-κB signaling is significantly upregulated both in the cerebral cortex and cerebellum in the *Npc1*^−/−^/*App*^−/−^ brain (Figure 8), we further speculate that NF-κB signaling may contribute to cholesterol dysregulation in these mice through its effect on RTKN2.

We have also described aberrant patterns of tau phosphorylation in both the cerebral cortex and cerebellum of *Npc1*^−/−^/*App*^−/−^ mice [9]. Remarkably, our present analysis shows enrichment of the tau protein kinase activity gene set only in response to APP loss in the cerebral cortex, both in NPC (*Npc1*^−/−^/*App*^−/−^) and in wildtype backgrounds (*App*^−/−^) (Figure 14A,C,D), but not in the cerebellum (Figure 15). Upon closer examination, we noticed that GSEA core enrichment consistently lists Phkg1 as the top gene involved in tau kinase activity within the *Npc1*^−/−^/*App*^−/−^ and *App*^−/−^ cerebral cortex (Figure 14A,C,D). Phosphorylase kinase γ subunit (Phkg1) is a serine/threonine kinase enzyme that is involved in various biological and signaling processes such as glycogenolysis, platelet activation, and neuronal function [21]. Phkg1 targets ser-262 in the tau protein [22]. This is significant for two reasons. First, ser-262 has been established as a priming site for tau phosphorylation, as inhibition of this single site prevents the appropriate regulation of tau phosphorylation [23]. Second, we have reported significant differences in phosphorylation rates at this site in our *Npc1*^−/−^/*App*^−/−^ mice when compared with *Npc1*^−/−^ and *App*^−/−^ mice [9]. We speculate that heightened activity of Phkg1 in the *Npc1*^−/−^/*App*^−/−^ mice could contribute to tau dyshomeostasis in the NPC cerebral cortex.

Analysis of the Regulation of Tau Protein Kinase Activity gene set shows a more complex outcome. As seen in Figure 16, this gene set is enriched in the cerebral cortex in *Npc1*^−/−^ mice compared with wildtype controls (Figure 16E), and this enrichment is exacerbated upon the loss of APP (*Npc1*^−/−^/*App*^−/−^; Figure 16A). However, there is no apparent APP-dependent enrichment of the Regulation of Tau Protein Kinase Activity gene set in the cerebellum (Figure 17). Our interpretation of these findings is that APP plays a role in regulating tau phosphorylation in the cerebral cortex, but its role in the cerebellum, if any, is not apparent from our analyses. Interestingly, a role for APP in regulating tau phosphorylation in the cerebral cortex has also been described in the context of AD [21], and this further suggests that the functional interaction between APP and tau may be more physiologically relevant in the cerebral cortex than in the cerebellum.

Finally, it is noteworthy that while the cGAS-STING pathway was shown to be activated in 8-week-old NPC mice [18], we did not find differences across Npc1 and App genotypes in the cerebellum or the cerebral cortex in our mice (Supplementary Figure S9). We interpret this apparent discrepancy to suggest that the cGAS-STING pathway is not activated before the symptomatic onset of NPC. Further research is needed to determine the relevance of this pathway to the pathogenesis of the disease.

## 5. Conclusions

**Figure 18:**
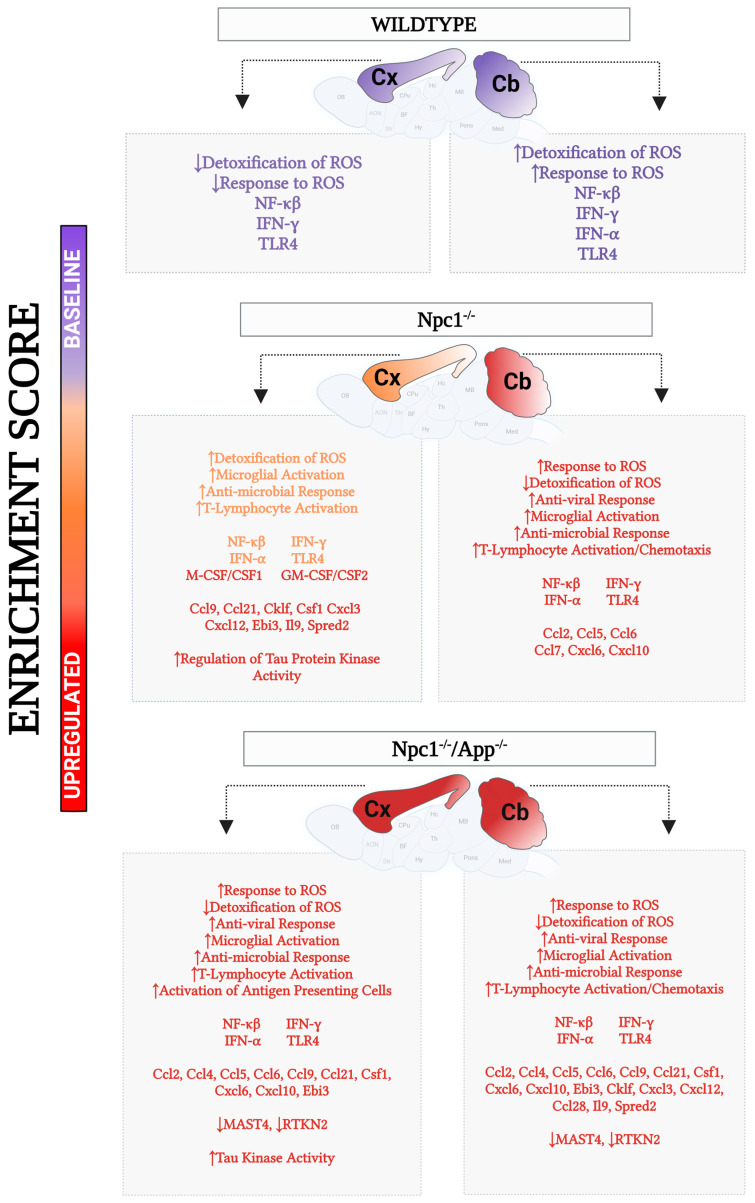
Summary of molecular and cellular changes identified in the cerebral cortex and cerebellum of 3-week-old mice of wildtype, *Npc1*^−/−^, and *Npc1*^−/−^/*App*^−/−^ genotypes. Up arrow = Activated/Upregulated. Down arrow = Inhibited/Downregulated.

Collectively, our findings, summarized in Figure 18, indicate that depletion of APP in the *Npc1*^−/−^ brain exacerbates pathology at multiple levels, both in the cerebellum and in the cerebral cortex. These results are entirely consistent with a hormetic cytoprotective role for APP in the central nervous system, as previously proposed by us [9,19]. Among all the genotypes examined, the *Npc1*^−/−^/*App*^−/−^ brain displayed the highest activation of IFN-γ and IFN-α pathways, alongside the highest enrichment of upstream regulators NF-κB and TLR4. Not surprisingly, APP/MAST4/RTKN2-driven regulation of cholesterol was also impaired in the *Npc1*^−/−^/*App*^−/−^ brain, given the likely contributory effects of NF-κB-driven changes on RTKN2 function. Downstream functional responses related to inflammation and oxidative stress were also more pronounced in the *Npc1*^−/−^/*App*^−/−^ brain when compared with *Npc1*^−/−^ and wildtype genotypes. Furthermore, and consistent with our previous work showing abnormal tau phosphorylation patterns at different neurodegeneration-linked phospho-epitopes [9], we now show enrichment patterns in tau kinase activity and regulation of tau kinase activity that help explain the differential effects of APP on tau phosphorylation in both the cerebral cortex and the cerebellum.

## Figures and Tables

**Table 1:** Differentially expressed genes identified in each genotype by genome-wide transcriptome analysis. All reported genotypes were compared against the wildtype. Significance is expressed by aFC > 1.5, *p* < 0.05. TR: number of transcript-reads by microarray; aFC: absolute fold change; DEG: differentially expressed gene. In this table, (*) indicates data that was not originally generated in this study. * The *Npc1*^−/−^ Crb vs. WT Crb and *Npc1^−/−^*App*^−/−^ Crb vs. WT Crb datasets were reported by Shin et al. [5]. The *Npc1^−/−^ vs. WT Ctx dataset was reported by Tolan et al. [11].

	Cortex			Cerebellum		
	*Npc1* ^−/−^	*App* ^−/−^	*Npc1*^−/−^ /*App* ^−/−^	*Npc1 ^−/−^	*App* ^−/−^	*Npc1 ^−/−^ /*App* ^−/−^
DEG (aFC + p)	321	843	1884	387	1065	1973
DEG (up)	199	459	1177	176	457	1265
DEG (down)	122	384	707	211	608	708

**Table 2:** Top eight predicted cytokine/chemokine upstream regulators across cerebral cortical genotypes, as determined by IPA upstream analysis. Enrichment Color Key: Red = Up. White = 0, Purple = Down, Black = Gene not listed in the dataset. #T.M (Target Molecules) = number of downstream target genes (DEGs) of each upstream regulator. In this table, (*) indicates data that was not originally generated in this study. *Npc1^−/−^ Ctx data originally reported in Tolan et al. [11].

Upstream Regulators	*Npc1*^−/−^/*App*^−/−^ Ctx vs. WT Ctx			*Npc1^−/−^ Ctx vs. WT Ctx			*App*^−/−^ Ctx vs. WT Ctx		
	z-Score	−log(p)	#T.M	z-Score	−log(p)	#T.M	z-Score	−log(p)	#T.M
IFN-γ	8.494	33.070	201	4.104	10.291	84	−0.844	3.650	66
M-CSF/CSF1	4.257	41.633	116	3.785	5.210	21	0.588	1.527	16
GM-CSF/CSF2	4.986	13.799	73	3.598	5.000	33	1.640	1.565	22
IL33	5.102	17.583	71	3.036	2.914	17	0.927	2.863	23
TNFα	6.099	29.403	238	2.908	8.028	96	−0.681	1.376	71
IL-4	3.152	47.044	258	2.902	5.775	57	0.838	3.440	55
IL-6	5.230	26.041	161	2.737	5.370	46	n/a		
IL-3	3.865	9.932	63	2.460	2.436	21	n/a		

**Table 3:** Top eight predicted cytokine/chemokine upstream regulators across cerebellar genotypes, as determined by IPA upstream analysis. Enrichment Color Key: Red = Up. White = 0, Purple = Down, Black = Gene not listed in the dataset. #T.M (Target Molecules) = number of downstream target genes (DEGs) for each upstream regulator. In this table, (*) indicates data that was not originally generated in this study. *Npc1^−/−^ Crb data originally reported in Tolan et al. [11]. *Npc1^−/−^/*App*^−/−^ Crb and *App^−/−^ Crb data reported in Shin et al. [5].

Upstream Regulators	*Npc1^−/−^/*App*^−/−^ Crb vs. WT Crb			*Npc1^−/−^ Crb vs. WT Crb			*App^−/−^ Crb vs. WT Crb		
	z-Score	−log(p)	#T.M	z-Score	−log(p)	#T.M	z-Score	−log(p)	#T.M
IFN-γ	9.324	38.497	262	5.432	21.225	84	−0.152	2.481	69
TNFα	6.724	17.390	258	4.694	12.412	81	−0.816	1.324	79
IFN-α (group)	6.567	14.712	84	2.981	11.699	33	−1.845	0	12
GM-CSF/CSF2	5.761	8.539	79	4.023	5.740	26	−0.239	2.119	28
IFN-β1	4.841	11.953	62	2.874	7.525	19	1.250	2.844	0
IL-1β	6.972	13.230	147	3.828	9.729	49	n/a		
IFN-α2	6.302	16.475	61	3.059	13.590	27	n/a		
IFN-β (group)	4.956	7.919	31	3.595	10.769	18	n/a		

**Table 4:** Downstream effects analysis of *Npc1*^−/−^ and *Npc1*^−/−^*App*^−/−^ mouse brain. Neuroinflammatory-related downstream functions were determined by IPA Disease and Function Analysis. The table shows the number of target molecules (#T.M) that contribute to the activation of each biological function. The *Npc1*^−/−^*App*^−/−^ cerebella contains more downstream DEGs across all functions when compared with the *Npc1*^−/−^ cerebella. The *Npc1*^−/−^*App*^−/−^ cerebral cortex also shows more downstream DEGs in every functional category, except for T-lymphocyte chemotaxis, when compared with the *Npc1*^−/−^ cerebral cortex. In this table, (*) indicates data that was not originally generated in this study. *Npc1^−/−^ Ctx data originally reported in Tolan et al. [11]. *Npc1^−/−^ Crb and *Npc1^−/−^/*App*^−/−^ Crb data reported in Shin et al. [5].

Function	*Npc1^−/−^ Crb	*Npc1^−/−^*App*^−/−^ Crb	*Npc1^−/−^ Ctx	*Npc1*^−/−^*App*^−/−^ Ctx
Microglial Activation	9	29	15	29
Antiviral Response	15	56	n/a	41
Antimicrobial Response	n/a	83	31	73
T-lymphocyte Activation	18	87	37	79
T-lymphocyte Chemotaxis	6	25	n/a	n/a
Activation of Antigen-Presenting Cells	n/a	87	43	95
Activation of Dendritic Cells	n/a	27	n/a	25

## Data Availability

The raw datasets that were used for the work presented in this article are not readily available because they are part of an ongoing study. Requests to access the datasets should be directed to S.S.

## Supplementary Materials

The following supporting information can be downloaded at: https://www.mdpi.com/article/10.3390/genes15081066/s1. Supplementary Table S1: Top enriched gene sets in the *Npc1*^−/−^*App*^−/−^ cerebral cortex using the GSEA Hallmark Database; Supplementary Table S2: IPA Disease and Functions in the *App*^−/−^ Cortex and Cerebellum; Supplementary Table S3: Differential expression of cytokine transcripts in the cerebral cortex vs. the cerebellum in the *Npc1*^−/−^, *Npc1*^−/−^, *App*^−/−^ and *App*^−/−^ mice; Supplementary Figure S1: Activation of IFN-γ-responsive genes in the *App*^−/−^ cerebral cortex; Supplementary Figure S2: Activation of IFN-γ-responsive genes in the *App*^−/−^ cerebellum; Supplementary Figure S3: Activation of IFN-α-responsive genes in the *App*^−/−^ cerebral cortex; Supplementary Figure S4: Activation of IFN-α-responsive genes in the *App*^−/−^ cerebellum; Supplementary Figure S5: GSEA Interferon and Oxidative Stress gene sets in the *App*^−/−^ cerebral cortex; Supplementary Figure S6: GSEA Interferon and Oxidative Stress gene sets in the *App*^−/−^ cerebellum; Supplementary Figure S7: IPA core analysis of the *App*^−/−^ cerebral cortex; Supplementary Figure S8: IPA core analysis of the *App*^−/−^ cerebellum; Supplementary Figure S9: IPA cGAS-STING analysis of the *Npc1*^−/−^ and *Npc1*^−/−^/*App*^−/−^ brain regions.

## References

[1] Burton B.K., Ellis A.G., Orr B., Chatlani S., Yoon K., Shoaff J.R., Gallo D. (2021). Estimating the prevalence of Niemann-Pick disease type C (NPC) in the United States. Mol. Genet. Metab..

[2] Yanjanin N.M., Vélez J.I., Gropman A., King K., Bianconi S.E., Conley S.K., Brewer C.C., Solomon B., Pavan W.J., Arcos-Burgos M. (2010). Linear clinical progression, independent of age of onset, in Niemann–Pick disease, type C. Am. J. Med. Genet. Part B Neuropsychiatr. Genet..

[3] Lloyd-Evans E., Morgan A.J., He X., Smith D.A., Elliot-Smith E., Sillence D.J., Churchill G.C., Schuchman E.H., Galione A., Platt F.M. (2008). Niemann-Pick disease type C1 is a sphingosine storage disease that causes deregulation of lysosomal calcium. Nat. Med..

[4] Shin S.D., Shin A., Mayagoitia K., Wilson C.G., Bellinger D.L., Soriano S. (2019). Interferon downstream signaling is activated early in pre-symptomatic Niemann-Pick disease type C. Neurosci. Lett..

[5] Shin S.D., Shin A., Mayagoitia K., Siebold L., Rubini M., Wilson C.G., Bellinger D.L., Soriano S. (2019). Loss of amyloid precursor protein exacerbates early inflammation in Niemann-Pick disease type C. J. Neuroinflammation.

[6] Ong W.Y., Kumar U., Switzer R.C., Sidhu A., Suresh G., Hu C.Y., Patel S.C. (2001). Neurodegeneration in Niemann-Pick type C disease mice. Exp. Brain Res..

[7] Bajwa H., Azhar W. (2024). Niemann-Pick Disease. StatPearls.

[8] Mengel E., Klünemann H.H., Lourenço C.M., Hendriksz C.J., Sedel F., Walterfang M., Kolb S.A. (2013). Niemann-Pick disease type C symptomatology: An expert-based clinical description. Orphanet J. Rare Dis..

[9] Nunes A., Pressey S.N.R., Cooper J.D., Soriano S. (2011). Loss of amyloid precursor protein in a mouse model of Niemann–Pick type C disease exacerbates its phenotype and disrupts tau homeostasis. Neurobiol. Dis..

[10] Kodam A., Maulik M., Peake K., Amritraj A., Vetrivel K.S., Thinakaran G., Vance J.E., Kar S. (2010). Altered levels and distribution of APP and its processing enzymes in Niemann-Pick Type C1-deficient mouse brains. Glia.

[11] Tolan A.J., Sanchez K.L., Shin S.D., White J.B., Currais A., Soriano-Castell D., Wilson C.G., Maher P., Soriano S. (2024). Differential Interferon Signaling Regulation and Oxidative Stress Responses in the Cerebral Cortex and Cerebellum Could Account for the Spatiotemporal Pattern of Neurodegeneration in Niemann–Pick Disease Type C. Genes.

[12] Gong T., Liu L., Jiang W., Zhou R. (2020). DAMP-sensing receptors in sterile inflammation and inflammatory diseases. Nat. Rev. Immunol..

[13] Ayala A., Muñoz M.F., Argüelles S. (2014). Lipid peroxidation: Production, metabolism, and signaling mechanisms of malondialdehyde and 4-hydroxy-2-nonenal. Oxid. Med. Cell. Longev..

[14] Zampieri S., Mellon S.H., Butters T.D., Nevyjel M., Covey D.F., Bembi B., Dardis A. (2009). Oxidative stress in *Npc1* deficient cells: Protective effect of allopregnanolone. J. Cell. Mol. Med..

[15] Kielian T. (2019). Lysosomal storage disorders: Pathology within the lysosome and beyond. J. Neurochem..

[16] Suzuki M., Sugimoto Y., Ohsaki Y., Ueno M., Kato S., Kitamura Y., Hosokawa H., Davies J.P., Ioannou Y.A., Vanier M.T. (2007). Endosomal Accumulation of Toll-Like Receptor 4 Causes Constitutive Secretion of Cytokines and Activation of Signal Transducers and Activators of Transcription in Niemann–Pick Disease Type C (NPC) Fibroblasts: A Potential Basis for Glial Cell Activation in the NPC Brain. J. Neurosci..

[17] Liu T., Zhang L., Joo D., Sun S.-C. (2017). NF-κB signaling in inflammation. Sig. Transduct. Target. Ther..

[18] Chu T.-T., Tu X., Yang K., Wu J., Repa J.J., Yan N. (2021). Tonic prime-boost of STING signaling mediates Niemann–Pick disease type C. Nature.

[19] Gongol B., Marin T.L., Jeppson J.D., Mayagoitia K., Shin S., Sanchez N., Kirsch W.M., Vinters H.V., Wilson C.G., Ghribi O. (2017). Cellular hormetic response to 27-hydroxycholesterol promotes neuroprotection through AICD induction of MAST4 abundance and kinase activity. Sci. Rep..

[20] Gong C.-X., Iqbal K. (2008). Hyperphosphorylation of Microtubule-Associated Protein Tau: A Promising Therapeutic Target for Alzheimer Disease. Curr. Med. Chem..

[21] Caillet-Boudin M.-L., Buée L., Sergeant N., Lefebvre B. (2015). Regulation of human MAPT gene expression. Mol. Neurodegener..

[22] Winchester J.S., Rouchka E.C., Rowland N.S., Rice N.A. (2007). In Silico characterization of phosphorylase kinase: Evidence for an alternate intronic polyadenylation site in PHKG1. Mol. Genet. Metab..

[23] Ando K., Maruko-Otake A., Ohtake Y., Hayashishita M., Sekiya M., Iijima K.M. (2016). Stabilization of Microtubule-Unbound Tau via Tau Phosphorylation at Ser262/356 by Par-1/MARK Contributes to Augmentation of AD-Related Phosphorylation and Aβ42-Induced Tau Toxicity. PLoS Genet..

