# Supplementary material for: A Potential Role for the Amyloid Precursor Protein in the Regulation of Interferon Signaling, Cholesterol Homeostasis, and Tau Phosphorylation in Niemann–Pick Disease Type C
Source: Genes (Basel). 2024 Aug 13;15(8):1066. doi: 10.3390/genes15081066 (PMC11354009; doi:10.3390/genes15081066)
Supplement: Supplementary file 1 [file genes-15-01066-s001.zip › genes-3118887-supplementary.pdf]

Supplementary Materials

Supplementary Table S1. H1-HALLMARK DATABASE				
Gene Sets	Size	ES	NES	FDR-q
HALLMARK_REACTIVE OXYGEN SPECIES PATHWAY	45	0.55	1.77	0.227
HALLMARK_INTERFERON GAMMA RESPONSE	178	0.60	1.68	0.214
HALLMARK_INTERFERON ALPHA RESPONSE	85	0.69	1.64	0.175
HALLMARK_E2F TARGETS	190	0.41	1.56	0.231
HALLMARK_G2M CHECKPOINT	182	0.38	1.51	0.264
HALLMARK_ALLOGRAFT REJECTION	184	0.44	1.47	0.288
HALLMARK_MYC TARGETS V2	58	0.31	1.42	0.287
HALLMARK_IL6 JAK STAT3 SIGNALING	85	0.46	1.44	0.271
HALLMARK_INFLAMMATORY RESPONSE	195	0.39	1.36	0.301
HALLMARK_IL2 STAT5 SIGNALING	191	0.36	1.35	0.283
HALLMARK_KRAS SIGNALING UP	189	0.31	1.33	0.289
HALLMARK_APOPTOSIS	159	0.30	1.24	0.387
HALLMARK_PI3K AKT MTOR SIGNALING	103	0.25	1.20	0.438
HALLMARK_TNFA SIGNALING VIA NFKB	192	0.34	1.16	0.492
HALLMARK_HALLMARK SPERMATOGENESIS	131	0.25	1.16	0.474
HALLMARK_XENOBIOTIC METABOLISM	191	0.27	1.13	0.515
HALLMARK_COMPLEMENT	181	0.27	1.09	0.569
HALLMARK_ANDROGEN RESPONSE	91	0.27	1.05	0.644
HALLMARK_DNA REPAIR	137	0.22	1.00	0.719
HALLMARK_COAGULATION	131	0.25	1.00	0.690

Supplementary Table S1. Top Enriched Gene Sets in the Npc1$^{-/-}$App$^{-/-}$ Cerebral Cortex using the GSEA Hallmark Database. Four pathways from the Hallmark Database were listed as enriched in the Npc1$^{-/-}$App$^{-/-}$ cerebral cortex when run against all cerebral genotypes. Significance is determined by the FDR-q<0.25

Supplementary Table S2						
**Functions**	**$App^{-/-}$ Cortex**			**$App^{-/-}$ Cerebellum**		
	**p-value**	**z-score**	**#T.M**	**p-value**	**z-score**	**#T.M**
Microglial Activation	n/a	n/a	n/a	n/a	n/a	n/a
Antiviral Response	n/a	n/a	n/a	n/a	n/a	n/a
Antimicrobial Response	0.025	0	21	n/a	n/a	n/a
T-lymphocyte Activation	n/a	n/a	n/a	0.00318	-2.099	34
T-lymphocyte Chemotaxis	n/a	n/a	n/a	n/a	n/a	n/a
Activation of Antigen Presenting Cells	0.00974	0.133	23	n/a	n/a	n/a
Activation of Dendritic Cells	n/a	n/a	n/a	n/a	n/a	n/a

Supplementary Table S2. IPA Disease and Functions in the *App*$^{-/-}$ Cortex and Cerebellum. Neuroinflammatory-related downstream functions were determined by *IPA Disease & Function Analysis*. The table shows the number of target molecules (#T.M) which contribute to the activation of each biological function.

Supplementary Table S3												
Cytokines	Cerebellum						Cortex					
	*Npc1$^{-/-}$		*Npc1$^{-/-}$App$^{-/-}$		App$^{-/-}$		*Npc1$^{-/-}$		Npc1$^{-/-}$App$^{-/-}$		App$^{-/-}$	
	FC	*p-value*	*FC*	*p-value*	*FC*	*p-value*	*FC*	*p-value*	*FC*	*p-value*	*FC*	*p-value*
Ccl2	3.286	0.026	6.656	4.43E-04	n/a	n/a	ns	ns	2.511	0.00103	n/a	n/a
Ccl4	ns	ns	9.504	3.09E-02	n/a	n/a	4.664	0.0489	8.838	0.00296	n/a	n/a
Ccl5	4.772	0.0152	8.006	5.55E-04	n/a	n/a	ns	ns	5.626	0.00541	n/a	n/a
Ccl6	4.237	0.0134	7.516	3.58E-04	n/a	n/a	ns	ns	3.058	0.0130	n/a	n/a
Ccl7	1.961	0.0299	3.125	9.05E-03	n/a	n/a	ns	ns	ns	ns	n/a	n/a
Ccl9	ns	ns	ns	ns	n/a	n/a	1.624	0.039	3.132	0.00215	1.775	0.016
Ccl21	ns	ns	2.110	2.17E-02	n/a	n/a	2.823	0.0105	120.004	0.00115	n/a	n/a
Cklf	ns	ns	1.554	3.52E-02	n/a	n/a	1.809	0.03	ns	ns	n/a	n/a
Csf1	ns	ns	1.626	4.39E-02	n/a	n/a	2.183	0.0139	2.630	0.00320	n/a	n/a
Cxcl3	ns	ns	Ns	ns	n/a	n/a	1.812	0.0386	ns	ns	n/a	n/a
Cxcl6	1.754	0.0413	ns	ns	-2.002	0.00258	ns	ns	4.449	0.0196	n/a	n/a
Cxcl10	11.722	0.017	37.067	2.01E-04	n/a	n/a	ns	ns	21.868	0.00173	n/a	n/a
Cxcl12	ns	ns	ns	ns	n/a	n/a	1.984	0.0276	ns	ns	n/a	n/a
Ebi3	ns	ns	3.560	3.30E-03	n/a	n/a	2.101	0.007	2.115	0.00753	n/a	n/a
Il9	ns	ns	ns	ns	n/a	n/a	1.964	0.0088	ns	ns	n/a	n/a
Spred2	ns	ns	ns	ns	n/a	n/a	1.826	0.0221	ns	ns	n/a	n/a
Ccl24	-3.755	0.035	-5.372	2.47E-03	n/a	n/a	-2.887	0.0492	-5.898	0.0244	n/a	n/a
Ccl28	ns	ns	ns	ns	n/a	n/a	-1.514	0.0287	ns	ns	n/a	n/a
Clcf1	-2.152	0.0017	n/a	n/a	n/a	n/a	ns	ns	ns	ns	n/a	n/a
Cmtm5	ns	ns	ns	ns	n/a	n/a	-1.696	0.0491	-1.933	0.0215	n/a	n/a
Il23a	ns	ns	ns	ns	n/a	n/a	-2.746	0.0139	-3.488	0.0205	n/a	n/a

Supplementary Table S3. Differential expression of cytokine transcripts in the cerebral cortex vs. the cerebellum in the Npc1-/-, Npc1-/-App-/- and App-/- mice.

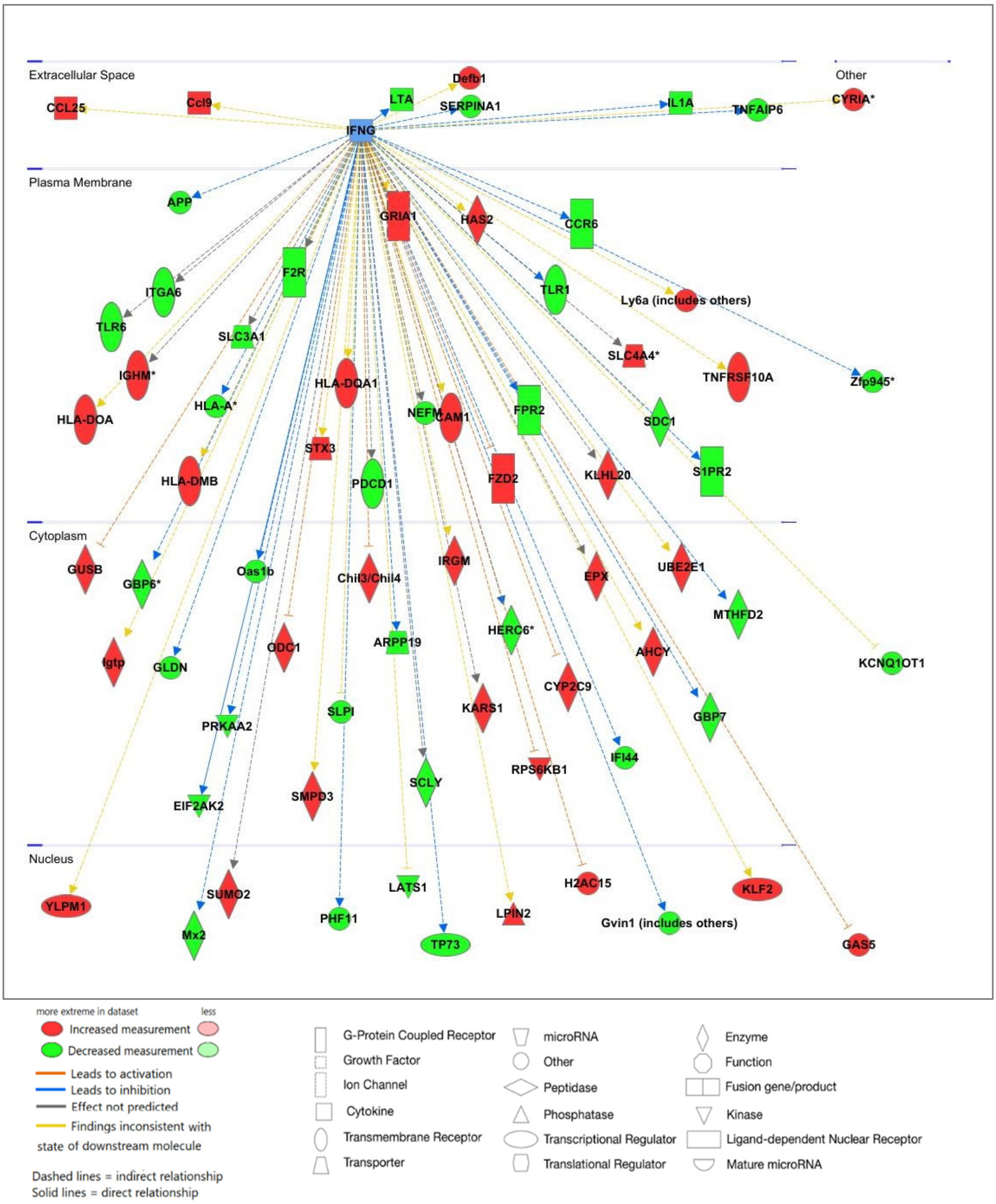

Supplementary Figure S1. Activation of IFN-γ responsive genes in the *App*$^{-/-}$Cerebral Cortex. 71 IFN-γ responsive genes are differentially expressed in the *App*$^{-/-}$ cerebral cortex compared with age-matched wild-type littermates. Of those, 35 DEGs are significantly upregulated and 36 are significantly downregulated. All DEGs are displayed in their sub-cellular location. All DEGS meet the significant criteria absolute fold change (aFC >1.5) and p-value (p<0.05).

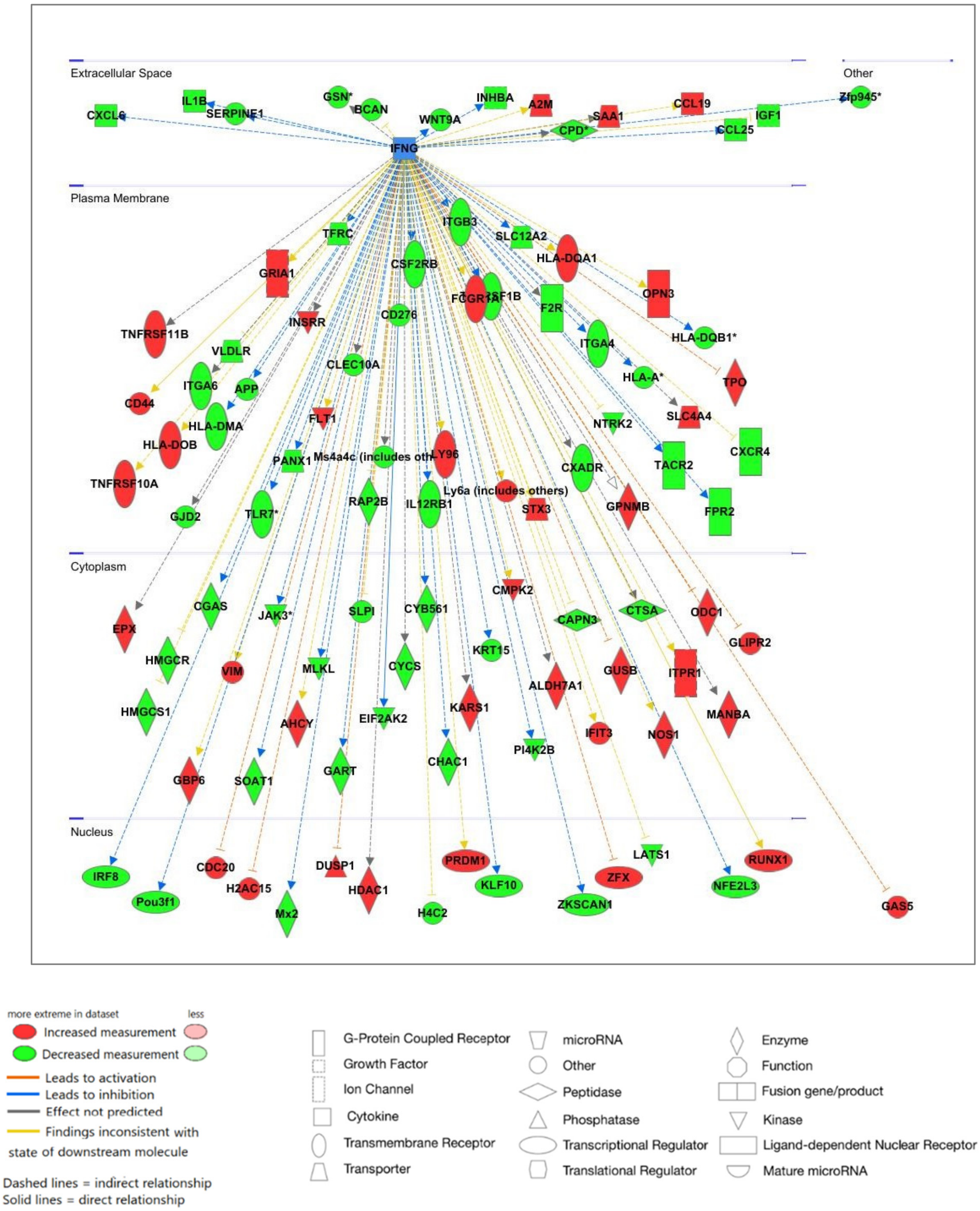

Supplementary Figure S2. Activation of IFN-γ Responsive Genes in the *App*$^{-/-}$ mouse cerebellum. 102 IFN-γ responsive genes are differentially expressed in the *App*$^{-/-}$ cerebellum compared with age-matched wild-type littermates. Of those, 41 DEGs are significantly upregulated and 61 are significantly downregulated. All DEGs are displayed in their sub-cellular location. All DEGS meet the significant criteria absolute fold change (aFC >1.5) and p-value ($p<0.05$).

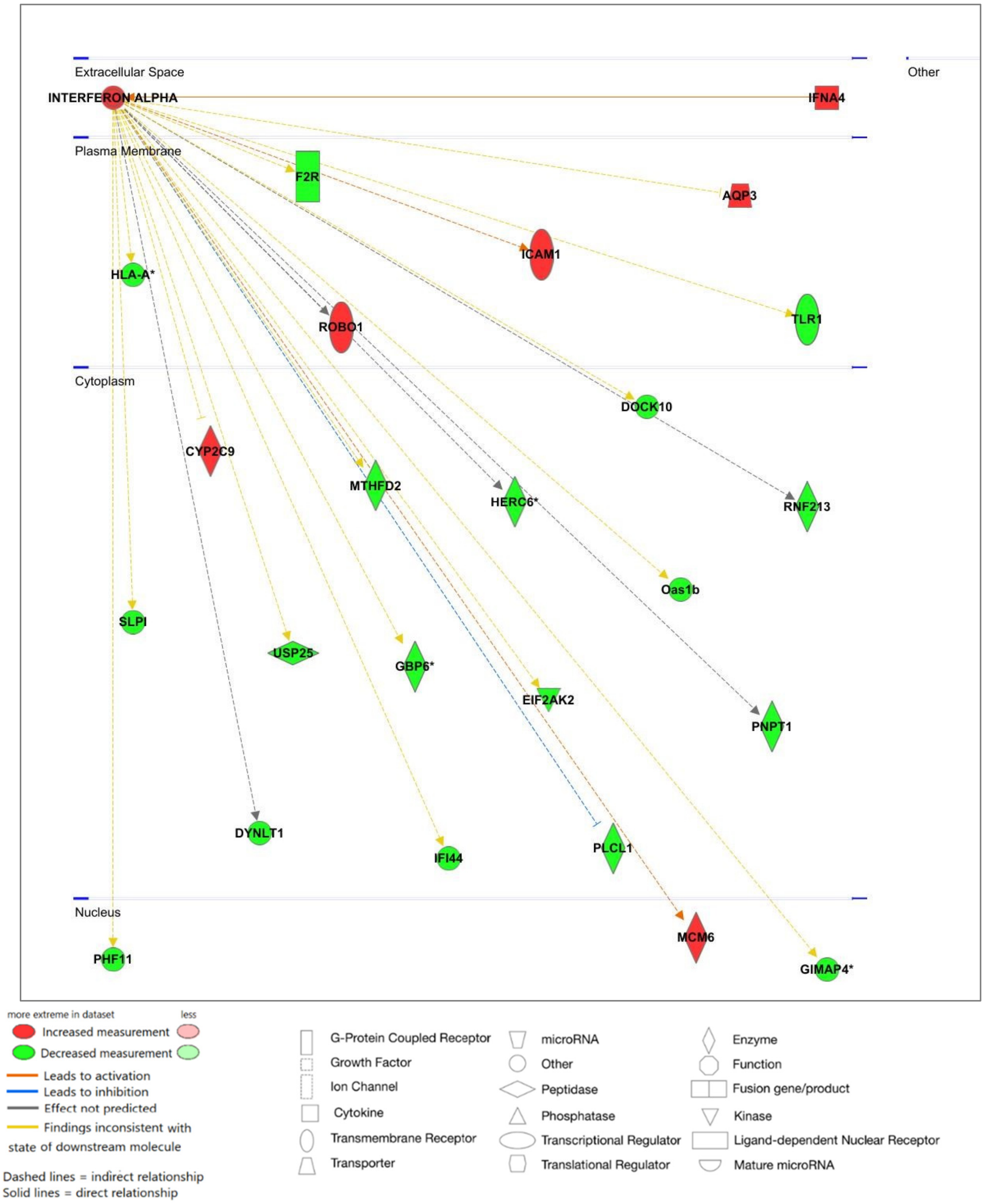

Supplementary Figure S3. Activation of IFN-α Responsive Genes in the *App*$^{-/-}$ Cerebral Cortex26 IFN-α responsive genes are differentially expressed in the *App*$^{-/-}$ cerebral cortex compared with age-matched wild-type littermates. Of those, 8 DEGs are significantly upregulated and 18 are significantly downregulated. All DEGs are displayed in their sub-cellular location. All DEGS meet the significant criteria absolute fold change (aFC >1.5) and p-value (p<0.05).

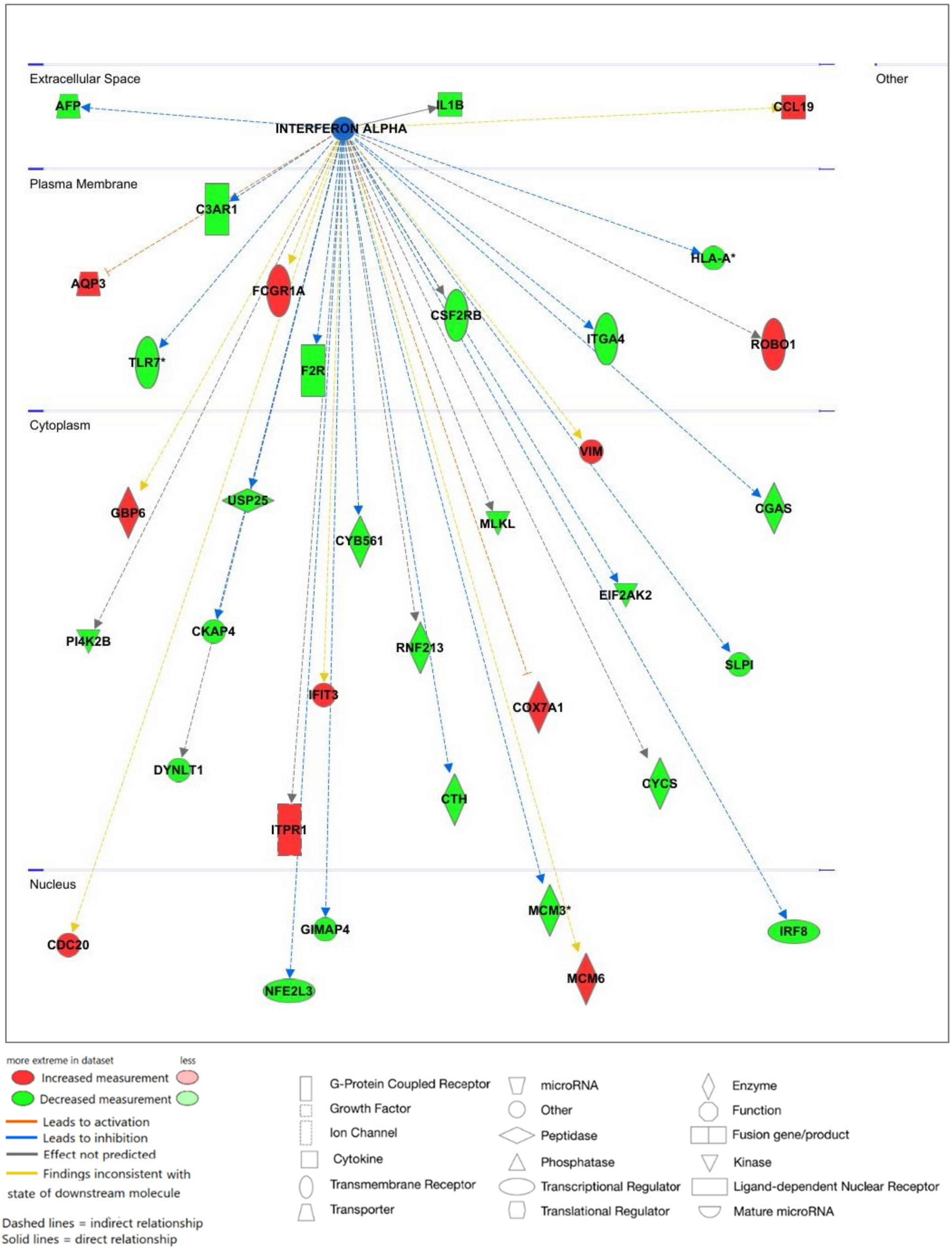

Supplementary Figure S4 Activation of IFN-α Responsive Genes in the *App*$^{-/-}$ Cerebellum. 35 IFN-α responsive genes are differentially expressed in the *App*$^{-/-}$ cerebellum compared with age-matched wild-type littermates. Of those, 11 DEGs are significantly upregulated and 24 are significantly downregulated. All DEGs are displayed in their sub-cellular location. All DEGS meet the significant criteria absolute fold change (aFC >1.5) and p-value (p<0.05).

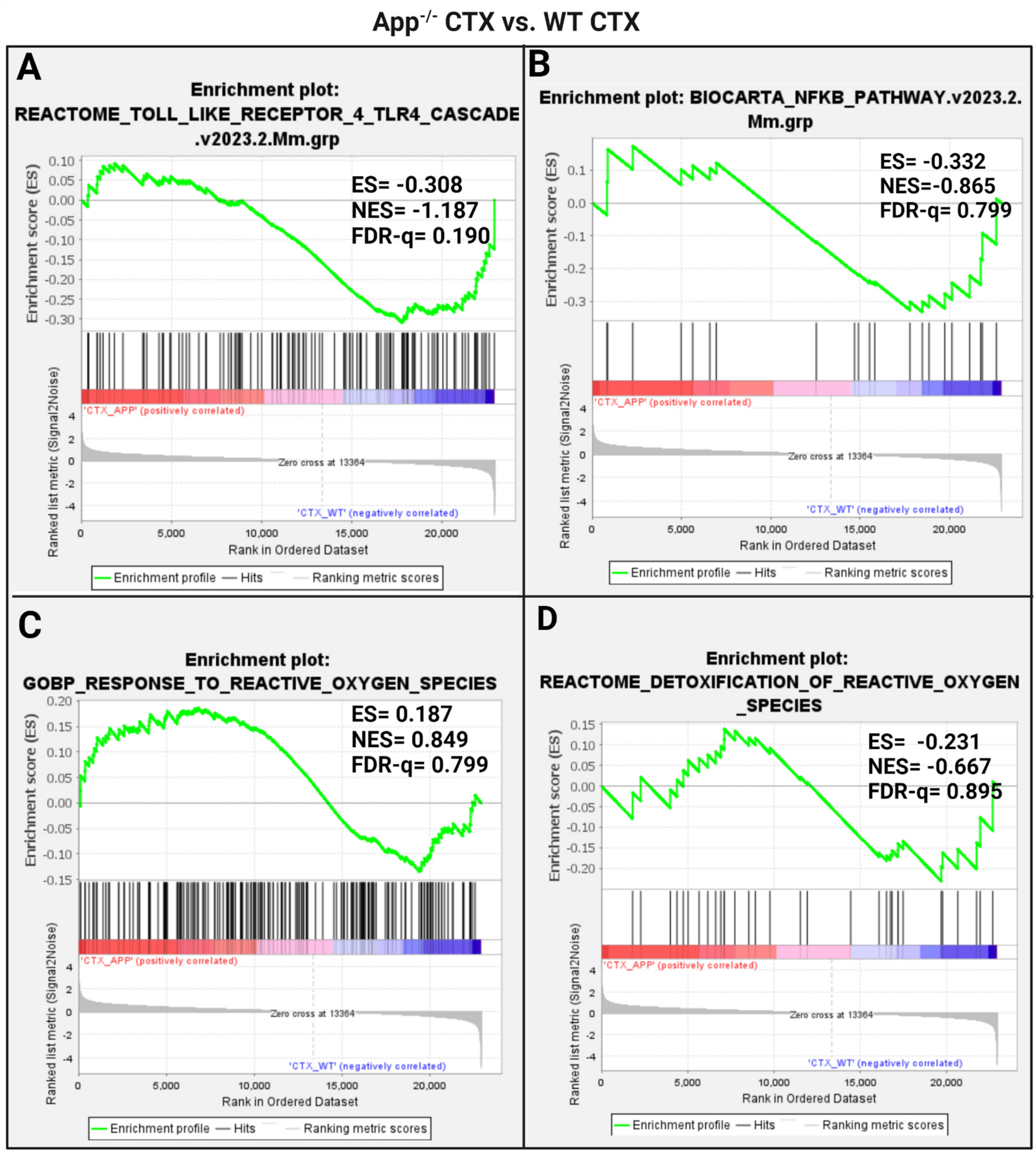

Supplementary Figure S5. GSEA Interferon and Oxidative Stress Gene Sets in the *App$^{-/-}$* Cerebral Cortex. The single App ko cerebral cortex showed no enrichment of upstream interferon regulators or oxidative stress pathways. Significant enrichment is determined by an FDR-q value of <0.25.

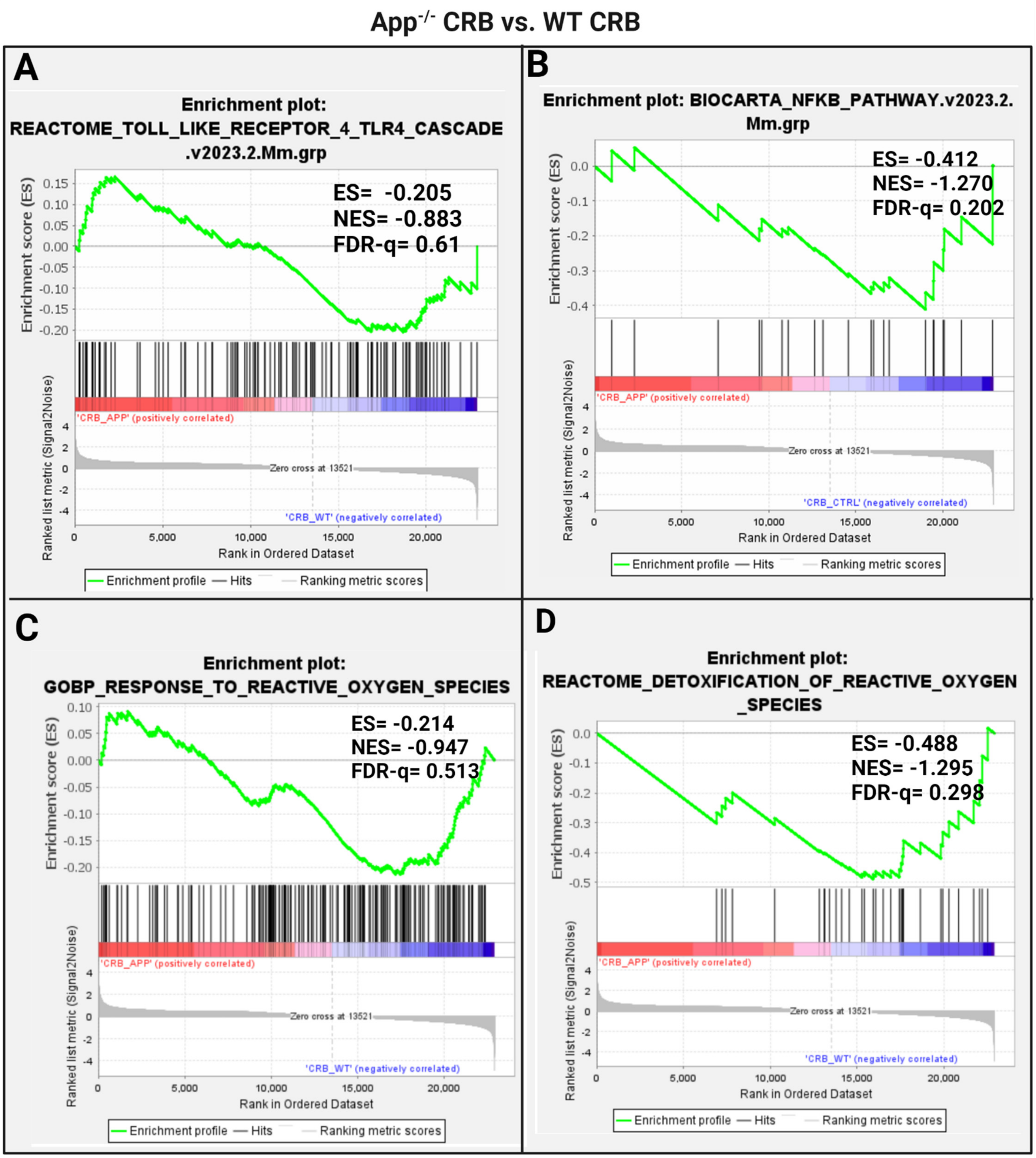

Supplementary Figure S6. GSEA Interferon and Oxidative Stress Gene Sets in the *App*$^{-/-}$ Cerebellum. The single App ko cerebellum showed no enrichment of TLR4 or oxidative stress pathways. NFkB was the only enriched upstream interferon regulator **(B).** Significant enrichment is determined by an FDR-q value of <0.25.

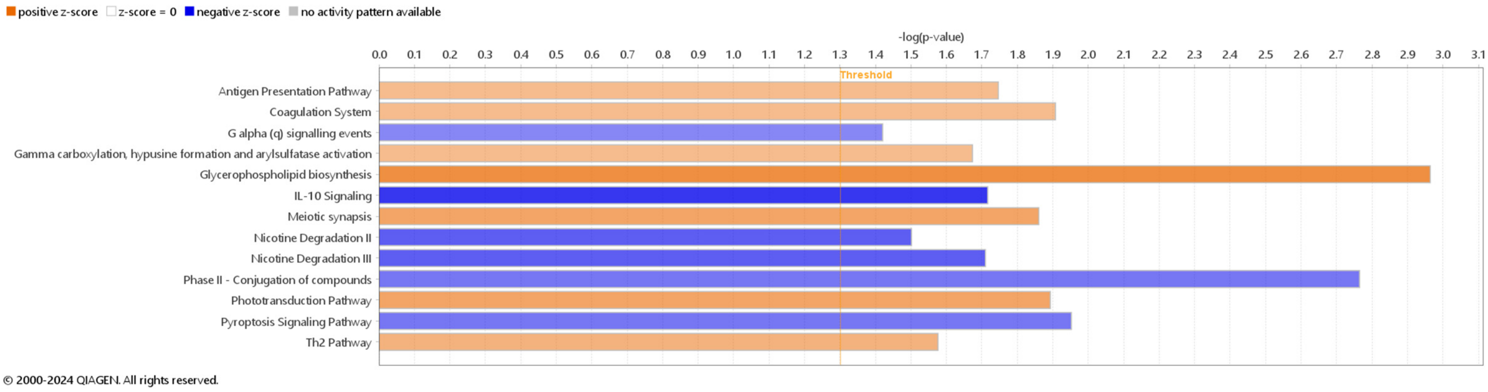

Supplementary Figure S7. IPA Core Analysis of the *App*$^{-/-}$ Cerebral Cortex. The single App ko cerebral cortex showed inhibition of IL-10 signaling, an anti-inflammatory pathway. Significance is determined by an absolute z-score >1 and p-value < 0.05.

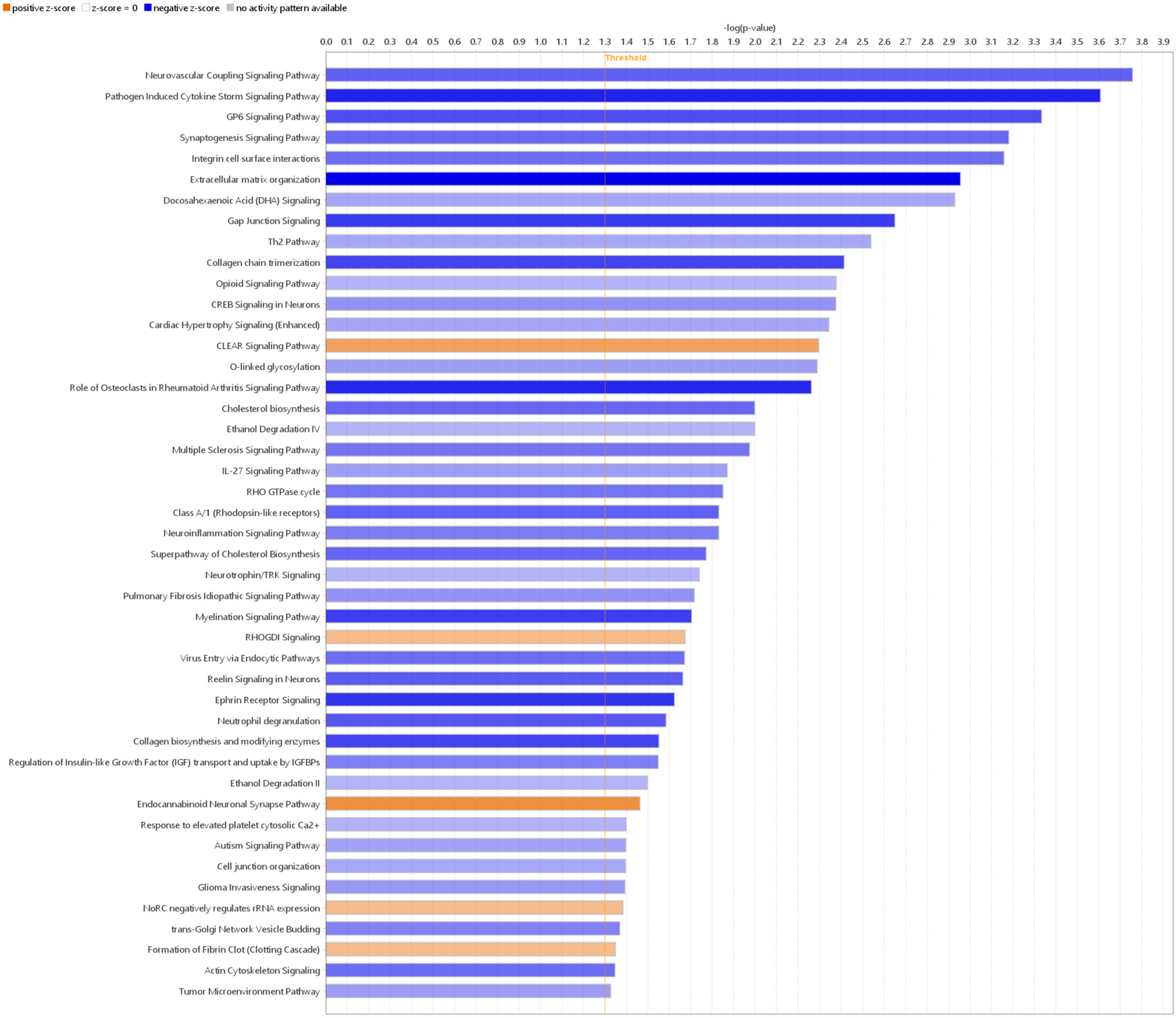

© 2000-2024 QIAGEN. All rights reserved.

Supplementary Figure S8. IPA Core Analysis of the *App*$^{-/-}$ Cerebellum. The single App ko cerebellum showed inhibition of many inflammatory pathways such as Pathogen Induced Cytokine Storm Signaling and Neuroinflammatory Signaling. Significance is determined by an absolute z-score >1 and p-value < 0.05.

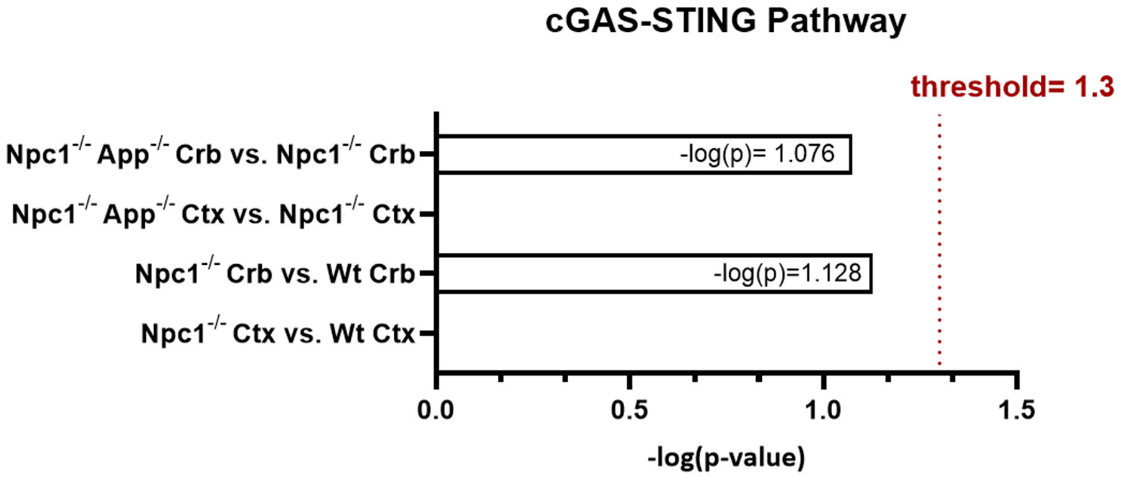

Supplementary Figure S9. IPA cGAS-STING Analysis of the Npc1$^{-/-}$ and Npc1$^{-/-}$/App$^{-/-}$ Brain Regions. There was no significant difference in cGAS-STING Pathway activation across genotypes and brain regions. cGAS-STING was undetectable in the *Npc1*$^{-/-}$*App*$^{-/-}$ vs. *Npc1*$^{-/-}$ cerebral cortex and *Npc1*$^{-/-}$ vs. wildtype cerebral cortex. Accordingly, no p-value was calculated.
